# Electron tomography reveals mitochondrial network and cristae remodelling during cell differentiation in the human placenta

**DOI:** 10.1038/s41467-026-75408-8

**Published:** 2026-07-08

**Authors:** Siddharth Acharya, Eric Hanssen, Veronica B. Botha, Tia M. Smith, Sahan Jayatissa, Zlatan Trifunovic, Lucy A. Bartho, John E. Schjenken, Tu’uhevaha J. Kaitu’u-Lino, Anthony V. Perkins, Joanna L. James, Kirsty G. Pringle, James C. Bouwer, Roger Smith, Joshua J. Fisher

**Affiliations:** 1 Mothers and Babies Research Centre, Hunter Medical Research Institute, New Lambton Heights, NSW Australia; 2 Reproductive and Family Health Research Program, Hunter Medical Research Institute, New Lambton Heights, NSW Australia; 3 School of Biomedical Sciences and Pharmacy, College of Health, Medicine and Wellbeing, University of Newcastle, Callaghan, NSW Australia; 4 Ian Holmes Imaging Centre and ARC Industrial Training Centre for Cryo-electron Microscopy of Membrane Proteins, The Bio21 Molecular Science and Biotechnology Institute, University of Melbourne, Parkville, VIC Australia; 5 Department of Biochemistry and Pharmacology, University of Melbourne, Parkville, VIC Australia; 6 Auckland Bioengineering Institute, University of Auckland, Auckland, New Zealand; 7 Translational Obstetrics Group, The Department of Obstetrics, Gynaecology and Newborn Health, Mercy Hospital for Women, University of Melbourne, Heidelberg, VIC Australia; 8 Centre for Reproductive Science, School of Science, College of Engineering, Science and Environment, University of Newcastle, Callaghan, NSW Australia; 9 School of Health, University of the Sunshine Coast, Sippy Downs, QLD Australia; 10 Department of Obstetrics, Gynaecology and Reproductive Sciences, Faculty of Medical and Health Sciences, University of Auckland, Auckland, New Zealand; 11 Molecular Horizons and School of Chemistry and Molecular Bioscience, University of Wollongong, Wollongong, NSW Australia; 12 ARC Industrial Transformation Training Centre for Cryo-electron Microscopy of Membrane Proteins, University of Wollongong, Wollongong, NSW Australia; 13 School of Medicine and Public Health, College of Health, Medicine and Wellbeing, University of Newcastle, Callaghan, NSW Australia

**Keywords:** Cryoelectron tomography, Cryoelectron microscopy, Differentiation

## Abstract

Mitochondria adapt their structure through fusion and fission, yet how their morphology and cristae architecture change as cells differentiate remains unclear. The human placenta is a valuable model for studying mitochondrial diversity within a single tissue. As epithelial trophoblast cells of the placenta differentiate, their accompanying mitochondria morphologically and functionally transform. We use array tomography to characterise mitochondrial volume and network complexity. Cryo-electron tomography reveals two distinct subpopulations of mitochondria within preparations enriched for progenitor cytotrophoblasts, and a singular, homogeneous population in the differentiated syncytiotrophoblast. We showcase the 3D topology of individual cristae through a standardised metric of “curvedness”. Proteomic analysis of isolated mitochondria identifies reduced levels of proteins involved in mitochondrial dynamics and cristae organisation complexes in the syncytiotrophoblast, accompanied by variations in electron transport chain subunits, ATP synthase, and supercomplex components. This study highlights the advantages of combining multimodal imaging with proteomic analyses, to elucidate the process of mitochondrial morphology and cristae architecture remodelling as cells differentiate.

## Abstract (web summary)

Mitochondria adapt their structure through fusion and fission. Here the authors couple array and cryo-electron tomography with proteomic analysis to descript changes in the mitochondrial network and cristate remodelling in progenitor and differentiated trophoblasts of the human placenta.

## Introduction

Mitochondria are intracellular organelles responsible for energy production, steroid hormone synthesis, and the regulation of cellular metabolism and immunity.^1^ Mitochondria produce adenosine triphosphate (ATP), facilitating cellular proliferation, migration, and the catalysis of biochemical reactions.^1^ Additionally, mitochondria adapt their bioenergetic capacity by remodelling their gross network structure through a continuous process of fusion and fission, concurrent with the remodelling of internal mitochondrial architecture.^2,3^ Despite the significance of mitochondrial structure and its role in dictating functional capacity, our understanding of the underlying protein mechanisms remains incomplete.

Mitochondrial fusion occurs when cellular demands for ATP are high, resulting in the formation of interconnected structures with an increased surface area and volume for efficient ATP synthesis^4^. This is achieved by mitofusin 2 (MFN2), which enables the fusion of the outer mitochondrial membrane, and optic atrophy 1 (OPA1), which promotes the fusion of the inner membrane.^2,5,6^ Conversely, when cellular demands are reduced, mitochondrial fission leads to the fragmentation of mitochondrial networks, an adaptation that supports basal ATP synthesis and specialised roles such as steroidogenesis and the clearance of dysfunctional mitochondria (mitophagy).^2,7–10^ Fission is driven by the presence of mitochondrial fission protein 1 (FIS1), which recruits dynamin-related protein 1 (DRP1) for cleavage.^5,11,12^ In certain cell types, fragmented mitochondria are physiologically beneficial, exhibiting a protective effect against the accumulation of reactive oxygen species and facilitating the distribution of mitochondria to distal regions of cells as observed in neuronal axons.^13,14^

Internal mitochondrial architecture is critical for regulating bioenergetic function and ATP synthesis efficiency. Cristae architecture is shaped by the mitochondrial intermembrane space bridging (MIB) complex, which comprises of the mitochondrial contact site and cristae organising system (MICOS), sorting and assembly machinery (SAM) complex, and metaxin proteins.^15^ The interaction between MIB complexes is critical for the organisation and structure of cristae, the formation of the intermembrane space, and the stability of inner membrane curvature.^15–17^ Conventionally, mitochondria within cardiac and skeletal muscle tissue contain stacked lamellar cristae, with the majority of the matrix occupied by closely packed, narrow folds with constricted crista junctions.^18,19^ In contrast, steroidogenic tissues such as the adrenal cortex and testis have mitochondria with rounded cristae, optimising the structure of the inner membrane for the import of cholesterol and subsequent synthesis of steroid hormones. The exact mechanism of interaction between MIB proteins and the formation of diverse cristae structures remains unclear; however, loss of cristae and inner membrane surface area is thought to limit the efficiency of the electron transport chain (ETC), a series of protein complexes that are critical for ATP synthesis.^20–23^

The ETC is comprised of complex I (NADH dehydrogenase), complex II (succinate dehydrogenase), complex III (Q-cytochrome c oxidoreductase), and complex IV (cytochrome c oxidase). Collectively, the complexes in mammalian mitochondria consist of at least 73 subunit proteins, which are typically categorised based on their function. Core subunits provide functional redox reaction centres for the transport of electrons, while accessory subunits provide structural stability and enable conformational changes to occur that assist in proton translocation.^24–31^ The ETC facilitates an electrochemical gradient, which permits the movement of protons through ATP synthase, providing sufficient kinetic energy for the phosphorylation of adenosine diphosphate into ATP.^32,33^ ATP synthase also has the capacity to oligomerise, optimising bioenergetic efficiency and driving structural stability, in addition to inducing inner membrane curvature to form sharp cristae apices and topologically complex ridges.^34–41^ Conversely, lack of ATP synthase dimers leads to the formation of atypical onion and globular cristae.^37,42,43^ ETC complexes have also been suggested to assemble into higher-order structures known as supercomplexes.^44^

Supercomplexes are highly efficient and supersede the kinetic and functional limits of individual complexes. Supercomplexes assemble in many forms, most commonly I + III + IV or III + IV, and may coexist simultaneously with individual complexes in a dynamic state of plasticity.^45^ Studies report the presence of supercomplexes when cellular bioenergetic demands are high, increasing the efficiency of ATP synthesis by forming tunnels for steady electron flux, instead of undergoing sequential complex-to-complex electron transport, which is rate-limited.^46,47^ The presence of ATP synthase dimers and supercomplexes have primarily been characterised in plant and mammalian species, and their role in human cell biology is not fully understood. There is limited consensus on whether membrane curvature is solely induced by the presence of ATP synthase oligomers and supercomplexes, or conversely, whether curved membranes stabilise and promote the formation of higher-order complex structures.^48,49^ Moreover, our understanding of the interplay between mitochondrial dynamics, cristae architecture, the incorporation of ETC complexes, and how the assembly of ATP synthase dimers and supercomplexes converge to remodel mitochondrial structure remains incomplete.

We propose that the placenta is a valuable model for studying mitochondrial structure within a singular tissue environment as it exhibits dramatic differences in mitochondrial morphology that can be visualised within neighbouring progenitor and differentiated cell populations. The placenta’s unique, branched villous structure supports diverse functions that sustain pregnancy.^50–52^ Villi are lined with a multicellular layer that consists of inner cytotrophoblast (CTB) cells, which fuse together and terminally differentiate into the outer, multinucleated syncytiotrophoblast (STB).^50^ In contrast to the proliferative CTB, the STB has been shown to have a lower capacity for producing ATP, an adaptation thought to be driven by its specialised roles in mediating biochemical reactions and synthesising steroid hormones critical for pregnancy.^53–57^

We adopted a multimodal imaging approach, using array tomography and cryo-electron tomography (cryo-ET) to assess the complex 3D structure of mitochondrial networks and the topology and curvature of cristae. Additionally, we characterised proteins involved in mitochondrial dynamics, the MIB complex, subunits of complexes I-IV and ATP synthase, and the abundance of supercomplexes in the human placenta. Together, this provides insights into the mechanisms underpinning the remodelling of mitochondrial structure, and how mitochondria adapt to support shifting cellular demands.

## Results

### Array tomography reveals the differences in mitochondrial network complexity between CTBs and the STB

**Fig. 1:**
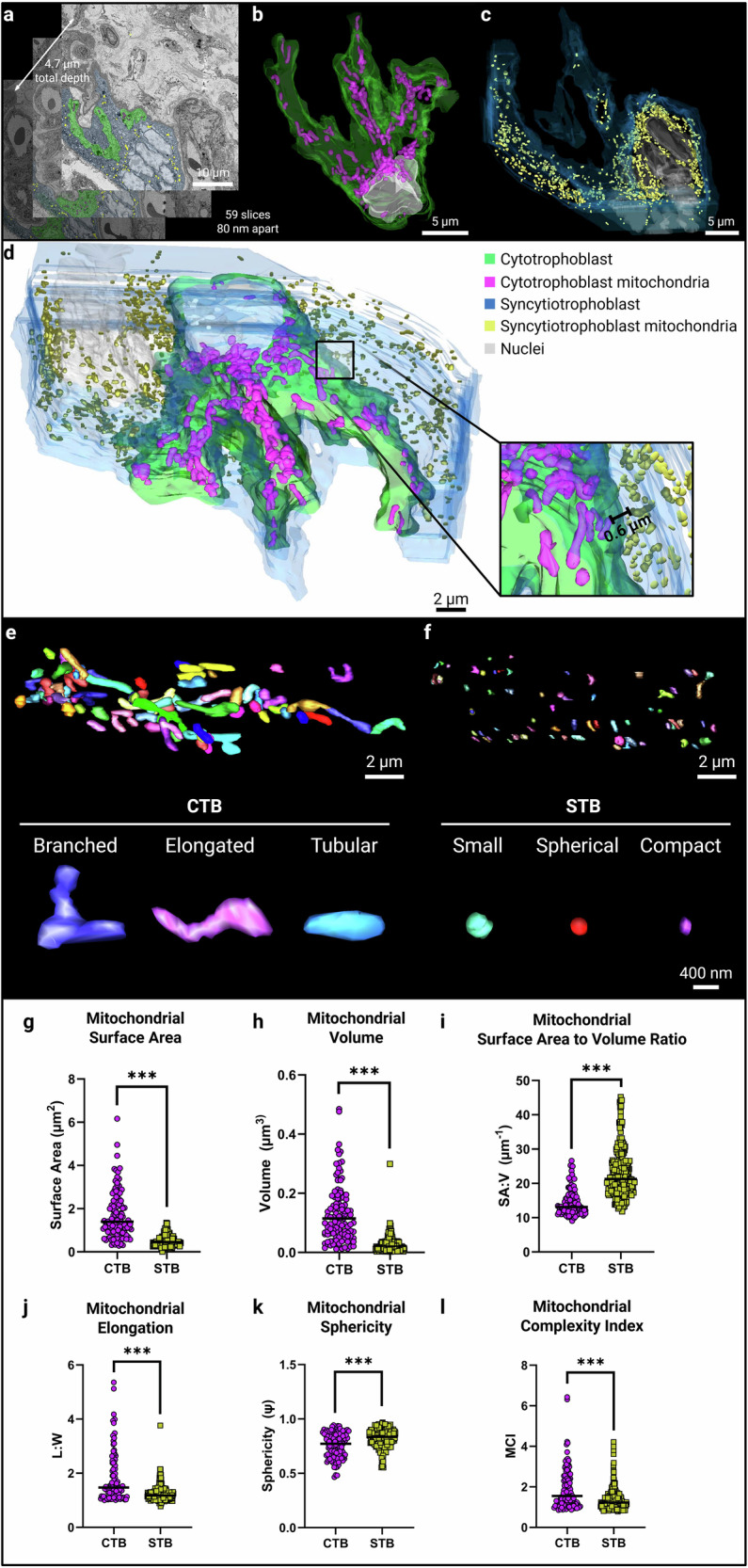
Array tomography of placental villi reveals mitochondrial networks of the CTB and STB. **a** Representative Z-slices from our array tomography image series with a Z-depth of 4.7 µm, the full image series had a total Z-depth of 20.16 µm. **b** 3D reconstruction of a CTB cell (green), nucleus (white), and CTB mitochondria (pink). **c** 3D reconstruction of the STB (blue), nucleus (white), and STB mitochondria (yellow). **d** Full in situ 3D reconstruction of a CTB and surrounding STB, revealing sub-micron distances between diverse mitochondrial structures. **e** Representative image of randomly selected subset of mitochondria from the CTB and **f** STB, both derived from one placental sample, with branched, elongated, and tubular mitochondria from the CTB and small, spherical, and compact mitochondria of the STB. **g**–**l** Volumetric analysis was performed on randomly selected subsets of mitochondria obtained from two CTBs (*n* = 113 Cyto-Mitochondria) and two STBs (*n* = 241 Syncytio-Mitochondria), collectively obtained from two healthy full-term placentae. The graphs presented are **g** surface area, **h** volume, **i** surface area to volume ratio, **j** elongation, **k** sphericity, and **l** mitochondrial complexity index. Two-sided linear mixed model analysis was performed to examine significant variations between groups, with cell population as a fixed effect, and the placental sample as a random intercept to account for clustering. Significant variation is expressed as *p* ≤ 0.001***. Created in BioRender. Pringle, K. (2026) https://biorender.com/m3o0ecx.

To characterise the 3D structure of whole mitochondrial networks within CTBs (Cyto-Mitochondria) or the STB (Syncytio-Mitochondria), we performed array tomography on resin-embedded villous tissue, derived from two healthy full-term placentae (Fig. 1a). Our reconstruction of the CTB and adjacent STB (Fig. 1b-d) showed the epithelial-like structure of the CTB, consisting of projections that surround the bordering basement membrane. Notably, despite being 0.6 µm apart, Cyto- and Syncytio-Mitochondria had contrasting network structures and morphologies. While Cyto-Mitochondria (pink) formed clusters and had a higher degree of interconnectedness, as reflected in their increased mitochondrial complexity index, Syncytio-Mitochondria (yellow) were fragmented and isolated (Fig. 1b-d, l). In addition to demonstrating contrasting gross network structures, our approach of reconstructing individual mitochondria built upon previously published 2D analyses,^53,55^ revealing a spectrum of morphologies including branched, elongated, and tubular mitochondria in the CTB, and small, spherical, and compact mitochondria in the STB (Fig. 1e, f). Cyto-Mitochondria were significantly larger and more elongated, with a mean surface area of 1.71 µm^2^ and volume of 0.135 µm^3^, compared to the smaller and more spherical Syncytio-Mitochondria, which had a mean surface area of 0.491 µm^2^ and volume of 0.031 µm^3^ (Fig. 1g, h, j, k). Consequently, Cyto-Mitochondria had a lower surface area to volume ratio (Fig. 1i).

### Transformation of mitochondrial cristae architecture following trophoblast differentiation

**Fig. 2:**
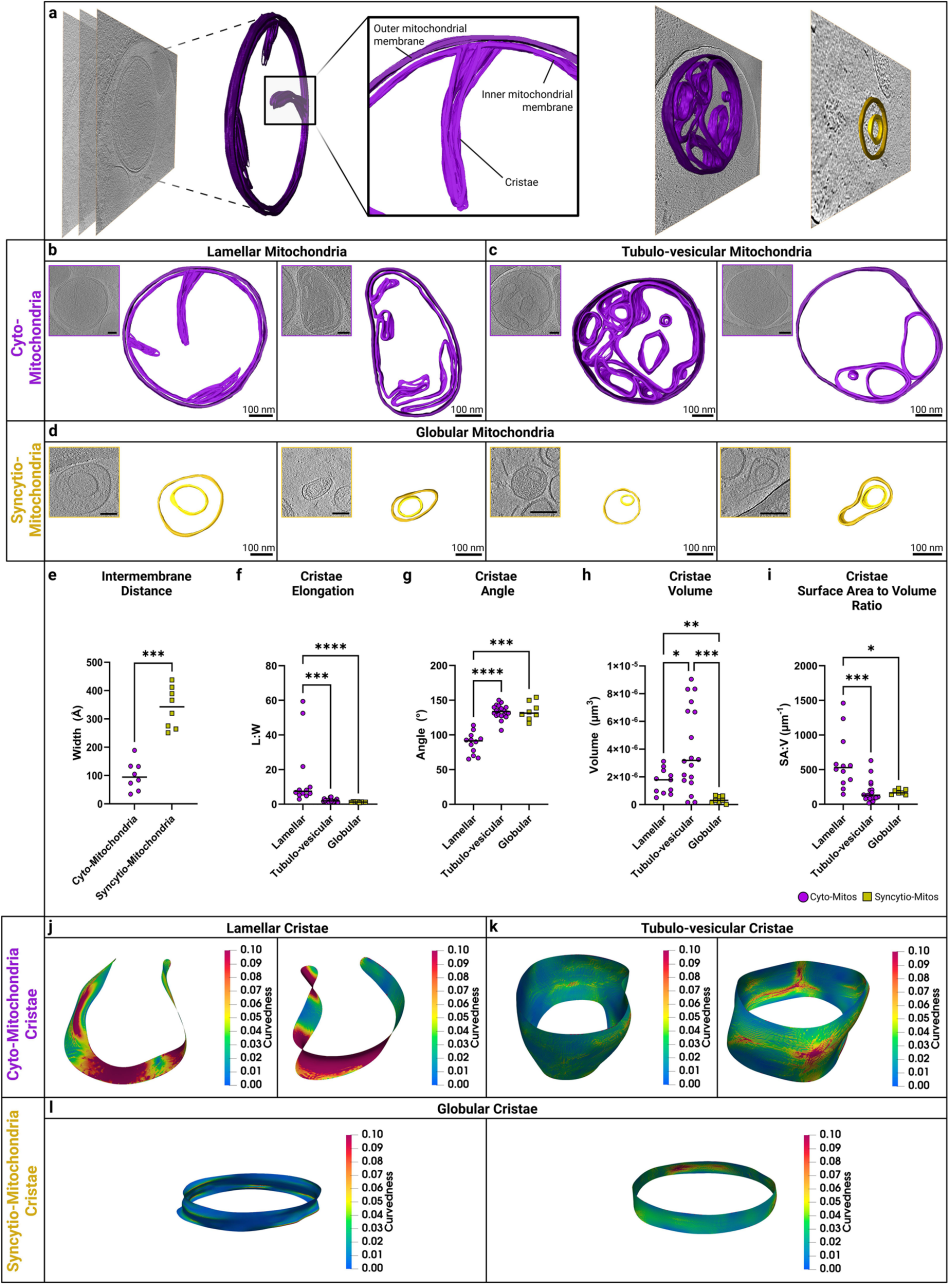
Cryo-ET analysis of internal mitochondrial architecture within preparations enriched for Cyto- and Syncytio-Mitochondria. **a** 16 mitochondria from two healthy full-term placentae were imaged, with representative tomographic slices of Cyto-Mitochondria (purple) and Syncytio-Mitochondria (yellow) shown. **b** Representative Cyto-Mitochondria that consist of lamellar cristae and **c** tubulo-vesicular cristae, and **d** representative Syncytio-Mitochondria that consist of globular cristae. All scalebars represent 100 nm. **e**–**i** Volumetric analyses were performed to quantify and compare cristae architecture between Cyto-Mitochondria (purple circles) and Syncytio-Mitochondria (yellow squares). **e** Intermembrane distance was compared between Cyto-Mitochondria and Syncytio-Mitochondria, whereby the width between the inner and outer mitochondrial membranes was measured across four quadrants, and the mean was plotted as a data point for each mitochondrion (*n* = 8 Cyto-Mitochondria, *n* = 8 Syncytio-Mitochondria). **f** Cristae elongation was assessed for each lamellar (*n* = 12 cristae), tubulo-vesicular (*n* = 19 cristae), and globular cristae (*n* = 8 cristae), between Cyto- and Syncytio-Mitochondria, where each data point represents an individual cristae. **g** Cristae angle was measured for lamellar (*n* = 12), tubulo-vesicular (*n* = 20), and globular (*n* = 8) cristae. Each data point represents the mean angle value of functionally pertinent zones of the cristae (apices and junctions for lamellar cristae, or using a quadrant system for tubulo-vesicular and globular cristae). Additionally, **h** cristae volume and **i** surface area to volume ratio were calculated using 3D reconstructions. Each data point represents an individual lamellar (*n* = 12 cristae), tubulo-vesicular (*n* = 19 cristae), and globular (*n* = 8 cristae) cristae. **j**–**l** 3D curvature was assessed across six cristae reconstructions, using the curvedness formula. Intermembrane distance was assessed using the Mann-Whitney t test, while volume was assessed using the Brown-Forsythe and Welch ANOVA test (post-hoc). All other cristae metrics were assessed using the Kruskal-Wallis with Dunn’s multiple comparisons tests (post-hoc). Significant variation is expressed as *p* < 0.05*, *p* ≤ 0.01**, *p* ≤ 0.001***, or *p* ≤ 0.0001****. Created in BioRender. Pringle, K. (2026) https://BioRender.com/pv5i14c.

Whilst array tomography enabled the observation of intact mitochondrial networks within their native tissue environment, its resolution and scale limited the investigation of internal mitochondrial membranes. Therefore, we used cryo-ET to increase the imaging resolution ~260-fold, to focus on imaging each individual mitochondrion within isolates enriched for Cyto- and Syncytio-Mitochondria as previously described, derived from two healthy full-term placentae.^53^ Following tomogram acquisition and 3D reconstruction of internal mitochondrial architecture (Fig. 2a), we observed a greater abundance of invaginated cristae in Cyto-Mitochondria, compared with Syncytio-Mitochondria (Fig. 2b-d). We observed two distinct mitochondrial subpopulations in Cyto-Mitochondria; those with lamellar cristae, formed by long inner membrane folds and sharp apex curvatures,^58^ and those with tubulo-vesicular cristae, which exhibited more rounded and bulbous structures.^19^ In contrast, Syncytio-Mitochondria only exhibited globular cristae architecture, characterised by rounded and spherical structures with no drastic deviations in curvature.^59^

Volumetric analyses of the cryo-ET data showed that Cyto-Mitochondria had a significantly smaller intermembrane distance than Syncytio-Mitochondria (Fig. 2e). Our analysis of individual cristae architectures revealed that lamellar cristae were significantly more elongated (Fig. 2f) and had a more acute degree of curvature at their functionally pertinent zones (apices and junctions) than either tubulo-vesicular or globular cristae (Fig. 2g). Within Cyto-Mitochondria, lamellar cristae had a lower volume than tubulo-vesicular cristae. However, both architectures had a greater volume compared to the smaller globular cristae of Syncytio-Mitochondria (Fig. 2h). Subsequently, we assessed surface area to volume ratio and determined that lamellar cristae of Cyto-Mitochondria had a significantly higher ratio than both tubulo-vesicular cristae and globular cristae (Fig. 2i). Building upon our 2D measurements, we characterised the complex topology and curvature of cristae by assessing the curvedness of the 3D cristae reconstructions, confirming that lamellar cristae had high curvedness localised to apices (Fig. 2j). Tubulo-vesicular cristae of Cyto-Mitochondria had uniformly distributed curvedness with localised pockets of angularity, while globular cristae of Syncytio-Mitochondria had the lowest curvedness compared to both subpopulations of Cyto-Mitochondria (Fig. 2k, l).

### Reduction in cristae organisation protein levels underpins the structural remodelling of syncytialised mitochondria

**Fig. 3:**
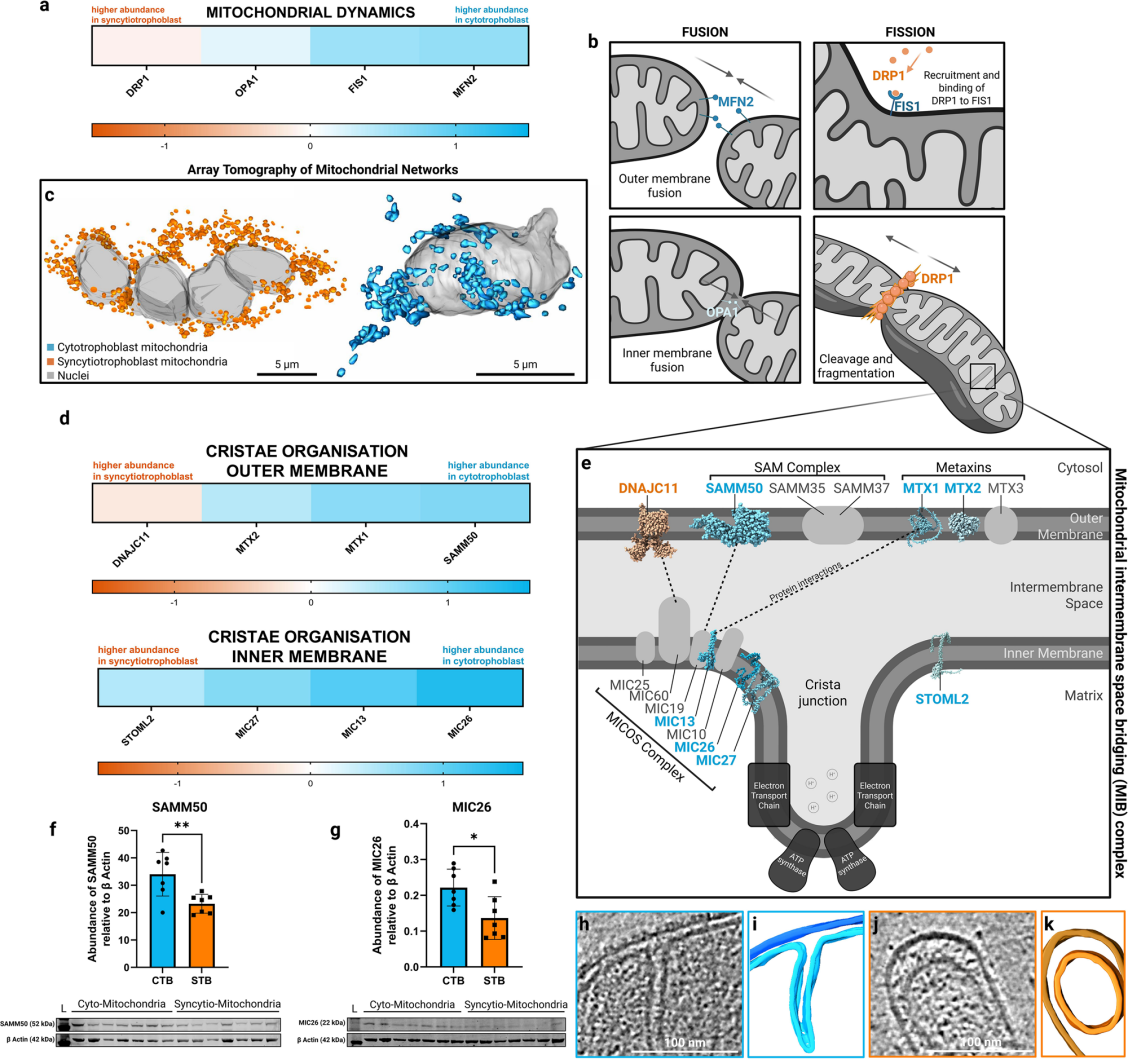
Proteomic analysis of Cyto- and Syncytio-Mitochondria enrichments to investigate the mechanisms involved in network fragmentation and structural remodelling. Heatmap graphs show the Log_2_ fold change (Log_2_FC) in protein abundance, with negative Log_2_FC indicating a higher abundance in Syncytio-Mitochondria enrichments (orange) and positive Log_2_FC indicating a higher abundance in Cyto-Mitochondria enrichments (blue). **a** Abundance of proteins involved in mitochondrial dynamics and network structure, including DRP1, OPA1, FIS1, and MFN2. **b**) Diagrammatic representation of mitochondrial dynamics proteins and their role in network fusion and fission of the outer and inner membranes. **c** Fusion of Cyto-Mitochondria (blue) and fragmentation of Syncytio-Mitochondria (orange) was confirmed by array tomography of placental villi. **d**) Abundance of proteins involved in the organisation of mitochondrial cristae, with the top row showing proteins embedded within the outer mitochondrial membrane, including DNAJC11, MTX2, MTX1, and SAMM50. The bottom row shows proteins embedded within the inner mitochondrial membrane, including STOML2, MIC27, MIC13, and MIC26. **e**) Diagrammatic representation of the mitochondrial intermembrane space bridging (MIB) complex, which includes protein complexes that are involved in organising mitochondrial cristae structure. Representative predicted protein structures have been shown, generated with Alphafold (DeepMind, UK).^104^ Western blotting was performed on mitochondrial isolates (*n* = 7 placentae) to validate the abundance of **f** SAMM50 (52 kDa), and **g** MIC26 (22 kDa), relative to β actin (42 kDa), between Cyto-Mitochondria (blue) and Syncytio-Mitochondria (orange) enrichments in a larger sample size. The molecular weight markers (L) shown on the left of each blot represent 50 kDa for SAMM50, 20 kDa for MIC26, and 40 kDa for β Actin. Data are presented as mean ± SD. Grubb’s outliers tests were performed on western blotting data, followed by two-sided unpaired t-tests to examine significant variation, expressed as *p* < 0.05*and *p* ≤ 0.01**. Blots shown below the graphs are representative of two independent experiments. **h**, **i** Images of Cyto-Mitochondria and **j**, **k** Syncytio-Mitochondria intermembrane spaces are shown, obtained from cryo-ET data. Created in BioRender. Pringle, K. (2026) https://BioRender.com/mwl33y1.

To understand the mechanisms underpinning the dramatic alterations in mitochondrial structure observed between Cyto- and Syncytio-Mitochondria following differentiation, we performed an in silico analysis of previously published liquid chromatography-mass spectrometry (LC-MS) data, obtained from mitochondrial isolates of three healthy full-term placentae.^57^ This enabled the examination of mitochondria-specific proteins that govern cristae architecture. When investigating the abundance of proteins involved in mitochondrial dynamics (the balanced interplay of fusion and fission), our findings indicated an increase in levels of fusion proteins MFN2 (0.64 Log_2_ fold change (Log_2_FC)) and OPA1 (0.16 Log_2_FC), and fission protein FIS1 (0.58 Log_2_FC) in Cyto-Mitochondria enrichments. Additionally, there was a higher abundance of the fragmentation and cleavage protein DRP1 (-0.12 Log_2_FC) in Syncytio-Mitochondria enrichments (Fig. 3a, b). Fragmentation of Syncytio-Mitochondria was confirmed by array tomography (Fig. 3c). Additionally, we investigated proteins of the MIB complex, including those within the SAM and MICOS complexes (Fig. 3d, e). We identified that proteins involved in connecting and tightening cristae junctions (including MTX2 (0.45 Log_2_FC), MTX1 (0.74 Log_2_FC), and SAMM50 (0.81 Log_2_FC)) were more abundant in Cyto-Mitochondria enrichments, whereas the inner membrane stability protein DNAJC11 (-0.21 Log_2_FC) was more abundant in Syncytio-Mitochondria enrichments. Within the MICOS complex, which has a critical role in maintaining the folding of mitochondrial cristae, we observed an increase of MIC27 (0.78 Log_2_FC), MIC13 (1.1 Log_2_FC), and MIC26 (1.4 Log_2_FC) in Cyto-Mitochondria compared to Syncytio-Mitochondria enrichments. Additionally, Cyto-Mitochondria enrichments had a higher abundance of the inner membrane stomatin-like protein 2 (STOML2, 0.49 Log_2_FC).

To further validate our proteomic analysis, we performed western blotting using mitochondrial isolates from an additional seven healthy full-term placentae, and characterised two MIB proteins, one located within the outer mitochondrial membrane (SAMM50) and the other residing within the inner mitochondrial membrane (MIC26), that had the greatest Log_2_FC between trophoblast populations. Given the role of SAMM50, MIC26, and the aforementioned proteins comprising the MIB complex in tightening the intermembrane space, we have included our cryo-ET 3D reconstructions in which we observed a greater intermembrane space in Syncytio-Mitochondria (Fig. 3j-k) compared to Cyto-Mitochondria (Fig. 3h-i).

### Subunit-specific variations in mitochondrial complexes between cyto- and syncytio-mitochondria enrichments

**Fig. 4:**
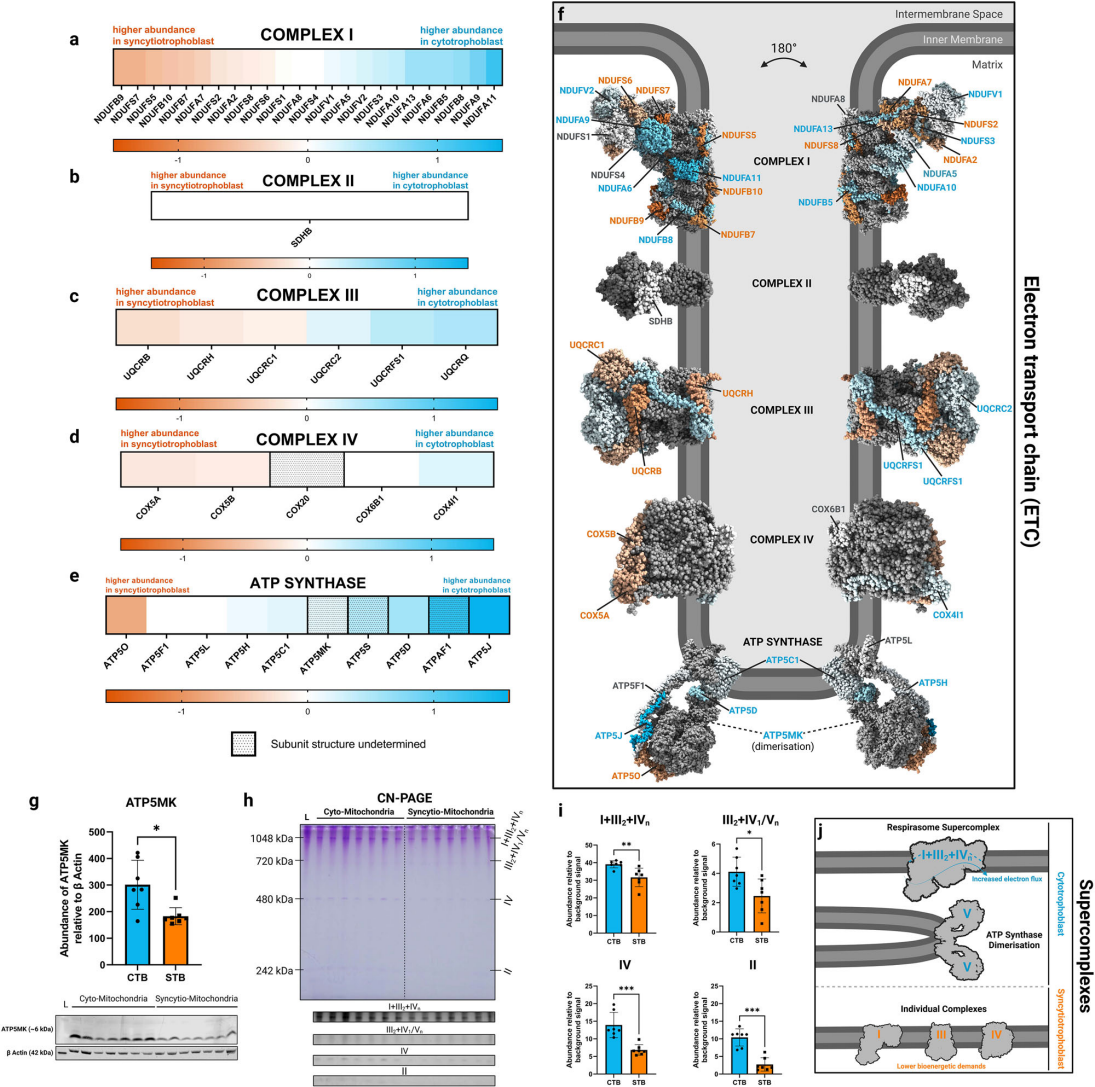
Proteomic analysis suggests altered ETC composition between the CTB and STB, to meet contrasting bioenergetic demands. Heatmap graphs show the Log_2_FC in protein abundance, with positive Log_2_FC indicating a higher abundance in Cyto-Mitochondria enrichments (blue) and negative Log_2_FC values indicating a higher abundance in Syncytio-Mitochondria enrichments (orange). Graphs show the abundance of protein subunits of **a** complex I, **b** complex II, **c** complex III, **d** complex IV, and **e** ATP synthase. Patterned heatmap squares (**c**, **d**) indicate subunits that have no solved structures in the representative models shown in (**f**). **f** Diagrammatic representation of the ETC and ATP synthase. As no protein models of placental mitochondria currently exist, representative cryo-EM models (obtained from HEK293F cells) have been used to show the location of each subunit, sourced from PDB (I: 5XTD, II: 8GS8, III: 5XTE, IV: 5Z62, V: 8H9V).^31,60–62 ^**g** Western blotting was performed using mitochondrial isolates (*n* = 7 placentae) to quantify the abundance of ATP5MK (7 kDa) relative to β actin (42 kDa), between CTBs (blue) and the STB (orange). The molecular weight markers on the left of each blot represent 10 kDa for ATP5MK and 40 kDa for β Actin. **h** CN-PAGE was performed using mitochondrial isolates (*n* = 7 placentae), and the representative membrane shows the abundances of supercomplexes I + III + IV (1048 kDa), III + IV, and individual ATP synthase (720 kDa), complex IV (480 kDa), and complex II (242 kDa). Quantitative analysis was performed on the excerpts of each band of proteins to produce graphs in (**i**). **j** Summary diagram showing that Cyto-Mitochondria have elevated levels of respirasome supercomplexes and ATP synthase dimerisation, while Syncytio-Mitochondria maintain individual complexes. The representative supercomplex diagram was produced as a silhouette of the cryo-EM model obtained from PDB (5XTH).^60^ Data are presented as mean ± SD. Grubb’s outliers tests were performed on western blotting data, followed by a two-sided Mann-Whitney U test to examine ATP5MK, and two-sided unpaired t-tests were used to examine CN-PAGE data. All blots are representative of two independent experiments. Significant variation is expressed as *p* < 0.05*, *p* ≤ 0.01**, *p* ≤ 0.001***, and *p* ≤ 0.0001****. Created in BioRender. Pringle, K. (2026) https://BioRender.com/mwl33y1.

To investigate the bidirectional relationship between cristae architecture and bioenergetic function, we assessed levels of ETC complex and ATP synthase subunit proteins. Our in silico analysis of the previously published proteomic data identified distinct subunit-specific variations in complexes I-IV and ATP synthase between Cyto- and Syncytio-Mitochondria enrichments. Specifically, we identified that complex I core subunits NDUFV1 (0.10 Log_2_FC), NDUFV2 (0.24 Log_2_FC), and NDUFS3 (0.30 Log_2_FC) were more abundant in Cyto-Mitochondria, whereas NDUFS8 (-0.17 Log_2_FC), NDUFS2 (-0.24 Log_2_FC), and NDUFS7 (-0.68 Log_2_FC) were more abundant in Syncytio-Mitochondria. Our analysis also showed a higher abundance of nine accessory subunits in Cyto-Mitochondria, while seven accessory subunits were more abundant in Syncytio-Mitochondria (Fig. 4a). While there were no changes in SDHB of complex II in our dataset (Fig. 4b), there was a higher abundance of three complex III subunits in Cyto-Mitochondria, and three other subunits were higher in Syncytio-Mitochondria (Fig. 4c). Our analysis of complex IV determined that only COX4I1 (0.21 Log_2_FC) was more abundant in Cyto-Mitochondria, whereas COX5B (−0.17 Log_2_FC) and COX5A (-0.21 Log_2_FC) were more abundant in Syncytio-Mitochondria (Fig. 4d). Investigation of ATP synthase subunits revealed that seven of the subunits were higher in Cyto-Mitochondria, while only ATP5O (−0.81 Log_2_FC) was higher in Syncytio-Mitochondria (Fig. 4e). To determine the position of each subunit identified in our proteomics analysis, subunit locations were mapped using representative protein structure models derived from HEK293 cells (Fig. 4f).^31,60–62^ Finally, to assess the capacity for ATP synthase dimerisation within Cyto- and Syncytio-Mitochondria enrichments, we confirmed by western blotting that Cyto-Mitochondria had a significantly higher abundance of dimerisation protein ATP5MK (0.19 Log_2_FC; Fig. 4g).

### Decreased supercomplex assembly in syncytio-mitochondria enrichments

Supercomplexes greatly increase kinetic efficiency and the capacity of mitochondria to synthesise ATP, compared to individual complexes. Clear native PAGE (CN-PAGE) was performed using mitochondrial isolates from an additional seven healthy full-term placentae, to determine differences in the abundance of supercomplexes between Cyto- and Syncytio-Mitochondria enrichments. Cyto-Mitochondria had a significantly higher abundance of supercomplexes I + III + IV (*p* = 0.0023) and III + IV (*p* = 0.0175), compared to Syncytio-Mitochondria. Cyto-Mitochondria also had a significantly higher abundance of complex IV (*p* = 0.0006) and complex II (*p* < 0.0006; Fig. 4h-j).

## Discussion

In this study, we conducted a multimodal 3D analysis of mitochondria from CTBs and the STB of the human placenta to study how changes in mitochondrial structure between differentiating cell types may translate to differences in function. Our imaging approach enabled both the investigation of mitochondrial networks in placental villous tissue in situ, and high-resolution analysis of inner membrane and cristae architecture in isolated human mitochondria. Using array tomography, we established that Cyto-Mitochondria have greater mitochondrial complexity and volume compared to differentiated Syncytio-Mitochondria. Within enriched mitochondrial isolates, cryo-ET revealed the internal architecture of Cyto- and Syncytio-Mitochondria, identifying that there are two unique subpopulations of mitochondria within progenitor cytotrophoblasts that have either lamellar or tubulo-vesicular cristae architecture. Notably, the architecture of both Cyto-Mitochondria subpopulations was distinct from Syncytio-Mitochondria, which only consisted of globular cristae. In turn, our proteomic analysis of enriched mitochondrial isolates identified that Syncytio-Mitochondria have a reduced capacity to undergo fusion and organise internal cristae architecture, with a decrease in MICOS and SAM complexes compared to Cyto-Mitochondria. These results occur in conjunction with alterations in specific core and accessory subunits across complexes I, III, and IV of the ETC, and ATP synthase. Moreover, we characterised the presence of supercomplexes in the placenta and observed that Syncytio-Mitochondria enrichments have decreased abundances of supercomplexes I + III + IV and III + IV. Collectively, our findings contribute to the broader understanding of mitochondrial physiology by linking alterations in cristae organisation proteins, dimerisation, and supercomplex abundance, with 2D angularity measures. These measures are supported by a standardised visual metric that accounts for 3D topology, thereby facilitating the comparison of localised cristae curvature (curvedness).

Our finding that Cyto-Mitochondria are larger and more elongated than Syncytio-Mitochondria confirms prior 2D observations of the structural diversity of mitochondria in the placenta.^55^ Our 3D mapping enables a more detailed analysis of the interconnected and complex network structures, demonstrating that Cyto-Mitochondria exhibit clustered and networked phenotypes. As mitochondrial networks are established via fusion, this structural pattern of larger, elongated, and more extensive networks is associated with the higher abundance of fusion proteins MFN2 and OPA1.^63^ In contrast, the smaller Syncytio-Mitochondria that exist in more fragmented networks are associated with an increase in DRP1.^11,12^ Indeed, the reduction of mitochondrial size following CTB differentiation into the STB shown in this study aligns with previous studies.^64^ The remodelling of mitochondrial networks between stem-like progenitors and differentiated progeny is well established in other tissues, and may similarly be critical for determining trophoblast fate in the placenta.^65^ Furthermore, mitochondrial fission may support steroidogenic function within the STB, facilitated by an increased mitochondrial surface area to volume ratio for the translocation of cholesterol to cytochrome P450, by which pregnenolone is synthesised.^55^ This is particularly necessary in the human placenta, given the lack of steroidogenic acute regulatory proteins that facilitate the translocation of cholesterol.^66–68^

In addition, we propose that fission may be necessary for mitochondrial distribution throughout the multinucleated STB, in a manner akin to neuronal axons; however, further analysis is required to assess links between spatial distribution and network structure.^14,69^ Furthermore, the presence of fragmented mitochondria in the STB is consistent with an increase in senescence-associated proteins in the STB such as P53, DCR2, and CDK inhibitors.^70,71^ Therefore, we suggest that in addition to functional specialisation, the fragmentation of the mitochondrial network may be a process of quality control and preparation for mitochondrial clearing (mitophagy) in the aging STB, and warrants further investigation.^8^ This finding was also supported by reduced levels of fusion proteins OPA1 and STOML2 in Syncytio-Mitochondria, proteins shown to promote inner membrane stability, and suggested to restrict mitophagy and promote mitochondrial biogenesis.^72,73^ As such, reduced STOML2 suggests that there is greater mitochondrial clearance in the STB.

We have shown that cristae from Syncytio-Mitochondria enrichments do not exhibit the deep invaginated structure that we observed in Cyto-Mitochondria, and also have an increased intermembrane space. Mechanistically, this may be explained by the concurrent loss of MICOS and SAMM proteins in Syncytio-Mitochondria, which would ordinarily increase cristae folding and subsequent bioenergetic capacity.^22,59^ Additionally, we propose that a greater intermembrane space in Syncytio-Mitochondria may facilitate more efficient diffusion of cholesterol to the inner mitochondrial membrane, where it is used by cytochrome P450 to synthesise pregnenolone.^55^ Given that cristae junctions serve as diffusion barriers by tightening the inner membrane and forming closed cristae pockets, the absence of observable cristae junctions in Syncytio-Mitochondria may permit greater accessibility for the transport of cholesterol to the inner membrane; however, further study is necessary to confirm this.^74,75^ In conjunction with the aforementioned STOML2, Syncytio-Mitochondria also had reduced levels of MTX proteins, which, although located within the outer mitochondrial membrane, are similarly critical for the stability of the cristae.^64,76,77^ In contrast, Cyto-Mitochondria had high levels of MTX and STOML2, in addition to high cristae curvatures, which we attribute to a greater abundance of supercomplexes and the potential for ATP synthase dimerisation as indicated by elevated ATP5MK within this study.^36,49,72^

Our examination of 3D cristae structure in the placenta enabled the identification of two unique subpopulations of Cyto-Mitochondria, comprising either lamellar cristae or tubulo-vesicular cristae architecture. Contrastingly, only a homogeneous population of globular cristae is present in the differentiated Syncytio-Mitochondria. Our findings demonstrate that lamellar cristae of Cyto-Mitochondria have a significantly higher surface area to volume ratio than the globular cristae of Syncytio-Mitochondria. The surface area to volume ratio of tubulo-vesicular cristae did not significantly differ from that of globular cristae. Our data suggest that the inclusion of lamellar cristae significantly elevates the exchange efficiency and functional capacity of the broader Cyto-Mitochondria population and, by extension, supports the higher bioenergetic demands of progenitor CTBs.^53,56–58^ The inverse relationship we observed between the outer and inner membrane surface areas is not only a hallmark of the invaginated structure of mitochondrial cristae, but would facilitate higher bioenergetic capacity through the provision of additional surface area for the placement of the ETC.^53,56,57^

To support and validate our 2D analysis of cristae angularity, we employed a standardised visual metric of curvedness that accounts for 3D topology and facilitates the comparison of localised cristae curvature. While previous studies have compared local cristae curvature within individual mitochondria, our approach enables the normalised comparison of multiple cristae from different mitochondrial populations. The acute angularity of lamellar apices and highly localised membrane curvedness we observed supports the greater bioenergetic capacity we and others have previously associated with Cyto-Mitochondria.^53,56,57^ Our findings are consistent with the theory that high curvature is induced by ATP synthase dimers, supercomplexes, OPA1, and MIB complexes, all observed in greater abundance within Cyto-Mitochondria enrichments.^21,22,34–36,59^ Globular cristae of Syncytio-Mitochondria had the lowest curvedness, consistent with lower potential for ATP synthase dimerisation and supercomplex abundance identified in this study.^53,56,57^ Given the role of cristae curvature in facilitating efficient proton funnelling through ATP synthase at apices, this data suggests a mechanistic basis for the remodelling of mitochondrial structure for shifting energetic requirements following trophoblast differentiation.^36,37,41,78^

In addition to the two distinct Cyto-Mitochondria subpopulations described, we observed a mitochondrion consisting of a combination of both lamellar and tubulo-vesicular cristae architectures (Supplementary Fig. 2). The co-existence of multiple cristae architectures within a singular mitochondrion has been suggested to enable a greater diversity of function in a variety of single-cell eukaryotes.^79^ Although singular in our dataset, the collective presence of at least two concurrent subpopulations of lamellar and tubulo-vesicular Cyto-Mitochondria poses the exciting prospect that we observed the process of “cristae differentiation”. As such, the development of mitochondrial subpopulations may precede, or accompany, the transition of proliferative CTBs to the previously identified pre-fusion CTBs in preparation for differentiation into STB.^80^ Our findings are aligned with the observations of *Ryu* et al.,^81^ who recently identified metabolically distinct mitochondrial subpopulations within mouse embryonic fibroblasts. Future work confirming the presence of mitochondrial subpopulations and distinct cristae architectures in situ is recommended and extensively discussed elsewhere.^82^

We characterised the ETC, revealing distinct variations in the composition of complexes between Cyto- and Syncytio-Mitochondria enrichments. While the abundance of core subunits within Complex I was varied equally between the populations, we found that most accessory subunits were more abundant in Cyto-Mitochondria. Since core subunits are necessary for electron transport and remain unchanged between cell populations, the loss of accessory subunits in Syncytio-Mitochondria suggests that there is a reduced dependence upon complex I structure, consistent with previous examinations of complex I and II linked respiration.^53^ Moreover, reductions in complex I accessory subunits have previously been shown to significantly impair the bioenergetic capacity of mitochondria within human embryonic kidney cells.^24^ Through a similar mechanism, the reduction in complex I accessory subunits in Syncytio-Mitochondria may be critical for the shift in cellular requirements following differentiation from CTBs.^24^ Although this study observed no change in complex II subunits, it has previously been identified that there is a lower abundance of SDHA in Syncytio-Mitochondria.^57^ Our findings are consistent with *Döhla* et al.’s work, which showed that mitochondria within stem-like progenitors support oxidative metabolism with a higher membrane potential, compared to newly synthesised mitochondria within differentiated cells.^83^ We suggest that altered metabolic characteristics of Syncytio-Mitochondria direct cell fate of the STB in a similar manner to that discussed by *Döhla* et al.; however, we are yet to assess whether selective mitochondrial inheritance is occurring alongside the withdrawal of mitochondrial proteins that govern the structure and efficiency of bioenergetic functions.^83^

The majority of ATP synthase subunits were higher in Cyto-Mitochondria enrichments. This finding is consistent with previously published results that showed greater abundance of ATP synthase subunits ATP5α and ATP5β in Cyto-Mitochondria.^57^ In addition, we observed a higher abundance of ATP5MK in Cyto-Mitochondria, which suggests a greater propensity for ATP synthase dimerisation in the CTB, a feature conserved across highly efficient bioenergetic mitochondria from yeast, bovine and human cells.^34,62,84^ Further, the increase in ATP5MK may contribute to the greater membrane curvature observed in Cyto-Mitochondria, with the assembly of ATP synthase dimers shown to induce membrane curvature, and localisation of electrochemical gradients at cristae apices.^36,40,85^ While not directly assessing dimerisation, our findings are consistent with the high curvedness of lamellar cristae apices, and acute angular pockets observed in tubulo-vesicular cristae in Cyto-Mitochondria relative to the globular cristae of Syncytio-Mitochondria.

In addition to ATP synthase dimers inducing curvature, cristae architecture has been proposed to be influenced by supercomplex assembly. It has been suggested that increased cristae curvature promotes the assembly of supercomplexes, through OPA1-linked mechanisms that contribute to internal architecture organisation.^86^ This concept is supported by our observation of increased OPA1 in Cyto-Mitochondria enrichments, in conjunction with greater membrane curvedness at cristae apices. Moreover, research has established that the placement of individual complexes may cause small deviations to the inner mitochondrial membrane, while rows of supercomplexes have been shown to significantly bend the inner membrane by up to 130°.^49^ The increased abundance of supercomplexes in Cyto-Mitochondria enrichments may explain the high curvedness in lamellar and tubulo-vesicular cristae within Cyto-Mitochondria, relative to Syncytio-Mitochondria. Moreover, the unexplained variations in ETC composition, particularly in accessory subunits, may promote greater assembly of the supercomplexes I + III + IV and III + IV observed in Cyto-Mitochondria. The subunit-specific variations we identified may also support the co-existence of supercomplexes and individual complexes in a dynamic state of plasticity.^45^ While supercomplexes in Cyto-Mitochondria may support increased bioenergetic demands and higher respiratory rates, reduced assembly in Syncytio-Mitochondria may explain previously reported lower demands for energy in the STB, and a shift in functionality from ATP synthesis towards steroidogenesis.^55,57^ In addition, we propose that the remodelling of mitochondrial structure following trophoblast differentiation is necessary to reduce the reliance of Syncytio-Mitochondria upon oxidative phosphorylation. This, in turn, limits the overutilisation of oxygen and substrates by Syncytio-Mitochondria, which would otherwise diffuse through the STB into fetal vessels, and support fetal development.

In this study, we showcased the advantage of using a multimodal imaging approach, exploiting the benefits of both array tomography and cryo-ET to study the diversity of mitochondrial structure in the placenta. While array tomography has the capacity to image whole and intact mitochondrial networks of the placenta, the technique is unable to be applied to study internal architecture and cristae structure due to resolution limits. This limitation is resolved by the incorporation of cryo-ET, which can acquire images with greater resolution and magnification. The mitochondrial isolation procedure used in our cryo-ET imaging has previously been validated to maintain mitochondrial capacity and respiratory function, assuring that mitochondria retain the functional characteristics of the specific trophoblast populations they are derived from, with up to 95% purity.^53,55,56^ While we acknowledge that a contribution from other villous cell types cannot be completely excluded without an exclusive marker for the cellular origin of isolated mitochondrial populations, further analysis of structural data demonstrates that Cyto- and Syncytio-Mitochondria within the enriched preparations retain the expected transverse diameter distributions of their in situ counterparts observed via array tomography, and are structurally divergent from endothelial and Hofbauer mitochondria (Supplementary Fig. 14). In the absence of an established methodology for interrogating high-resolution in situ mitochondrial network structure within specific trophoblast populations of placental tissue, we provide a solution by incorporating array tomography to characterise in situ mitochondrial structure alongside our high-resolution cryo-ET analysis.^53,82^ While our electron microscopy analysis was limited by the number of patient samples (*n* = 2 placentae for array tomography and *n* = 2 placentae for cryo-ET), we increased the number of mitochondria analysed, making statistical comparisons between mitochondria of the two cell populations (*n* = 354 mitochondria for array tomography and n = 16 mitochondria for cryo-ET), rather than assessing interpatient differences that would be used to investigate pathological instead of healthy mitochondrial structure.

To support our imaging observations, we conducted an in silico analysis within a previously published shotgun proteomics dataset,^57^ in which we compare protein abundance across specific pathways including MIB and ETC complexes. This enabled the comparison of Log_2_FC, which provides important insights into differences between Cyto- and Syncytio-Mitochondria enrichments. While statistical analysis of these differences was not performed in this study, we validated and confirmed the major findings from the proteomics data by performing western blotting on an additional seven placentae. CN-PAGE was performed to assess supercomplex abundance; however, this technique has a lower separation resolution than the alternative blue native PAGE (BN-PAGE) technique. However, we identified that the addition of Coomassie dye (a requirement of BN-PAGE) interfered with the separation of Cyto- and Syncytio-Mitochondria native complexes—a notable advantage of CN-PAGE.^87^

The placenta is a unique model for studying diverse mitochondrial structures within a single, bioenergetically dynamic organ. We have shown that progenitor CTBs adopt a more complex network of large, elongated, and branched mitochondria, which transition into fragmented, spherical, and compact mitochondria that are distributed across the multinucleated STB. We attribute the remodelling of the mitochondrial network to the reduction of fusion proteins MFN2 and OPA1, and an increase in fission protein DRP1 in Syncytio-Mitochondria enrichments. The reduction of cristae organisation proteins (MIB) in Syncytio-Mitochondria enrichments supports our cryo-ET observations of isolated mitochondria, in which we observed the absence of invaginated, lamellar cristae, and the formation of globular cristae in Syncytio-Mitochondria. Our findings suggest that these dramatic differences in morphology and cristae structure are in part influenced by alterations to core and accessory subunits of the ETC, impacting the ability of ATP synthase to form dimers and the assembly of supercomplexes. In summary, this study demonstrates the advantages of adopting a multimodal approach of array tomography and cryo-ET imaging in combination with proteomic analysis, to study diverse mitochondria and the mechanisms that link structure and function.

## Methods

### Ethical approval

Ethical approval was granted by the University of Newcastle Research Ethics Committee (H-382-0602), Hunter New England Health Human Research Ethics Committee (02/06/12/.13), Mercy Health Human Research Ethics Committee (R11/34), Griffith University Human Research Ethics Committee (MSC/05/15HREC), Queensland Government Human Research Ethics Committee (HREC/14/QPCH/246), and Site Specific Assessment (SSA/15/HNE/291) in compliance with the Declaration of Helsinki standards. With informed consent, placental samples were collected from healthy full-term pregnancies (38-40 weeks) at the John Hunter Hospital (NSW, Australia) and Mercy Hospital for Women (VIC, Australia). All reagents were sourced from Sigma-Aldrich, Australia, unless otherwise specified.

### Array tomography tissue preparation

Placental villous tissue was collected using a 6 mm biopsy punch from placentae of two healthy full-term pregnancies. Tissue samples were first washed in ice-cold PBS, then fixed in a solution of 1% (w/v) glutaraldehyde, 1.5% paraformaldehyde, 5 mM calcium chloride in 0.1 M sodium cacodylate. Samples were then prepared using the previously published reduced osmium fixation (ROTO) technique^88^ and then embedded in Procure 812 resin (ProSciTech, Australia).

### Array tomography image acquisition and processing

Resin-embedded placental tissue was serially sectioned at a thickness of 80 nm, using a Leica UC7 ultramicrotome (Leica Biosystems, Germany), and recovered onto silicon wafers that were sputter coated with 10 nm of carbon. Sections were then imaged using a FEI Teneo VolumeScope scanning electron microscope (Thermo Fisher Scientific, USA), with a voltage of 3 kV and current of 0.2 nA, acquired using MAPS software (Thermo Fisher Scientific, USA). Original images had resolution of either 6 or 8 nm, and were subsequently binned to 16 nm or 24 nm respectively, to improve the signal to noise ratio. Image series were then realigned using the IMOD package ETomo interface (University of Colorado, USA).^89^

### Array tomography image analysis and segmentation

Image series were imported into the IMOD software suite (University of Colorado, USA), and manual segmentation was performed on CTB membranes, nuclei, and arbitrary boundaries of STB of approximately equivalent volume to the CTB – due to the continuous nature of the STB. CTBs were identified based on their morphology and proximity to the STB, while the STB was identified by its distinct multinucleated morphology and microvilli.^52,90,91^ Mitochondria were identified for segmentation based on the electron density resulting from the double membrane (Supplementary Fig. 1). To investigate and quantify the structure of mitochondrial networks, all mitochondria within each cell type were initially segmented as a single object mesh. Subsequently, to characterise volumetric properties of each mitochondrion, four randomly selected Z-slices per cell type were selected, and all mitochondria within each selected slice were manually segmented as new individual object meshes (henceforth referred to as the ‘subset’). Mitochondria with obscured views or partial volumes were excluded from analyses. Each mitochondrion of the subset was meshed to form a 3D reconstruction, resulting in a subset of Cyto-Mitochondria (*n* = 113) and Syncytio-Mitochondria (*n* = 241). Elongation in the XY plane was calculated as a ratio from manual 2D measurements of maximum length and width. The sphericity (ψ) of each mitochondrion was calculated using the published formula^92,93^

1 $$\psi=\frac{{\pi }^{1/3}{(6V)}^{2/3}}{{SA}}$$

where *V* is volume and *SA* is surface area.

Additionally, we applied the previously published mitochondrial complexity index (MCI) formula,^94^ to quantify the structural complexity of each mitochondrion, as a method of investigating the degree of network interconnectedness and mitochondrial fusion occurring with neighbouring mitochondria

2 $${MCI}=\,\frac{{{SA}}^{3}}{16{\pi }^{2}{V}^{2}}$$

as previously described.^94^ While sphericity asymptotically reaches a value of one for a perfect sphere, MCI does not have theoretical limits—allowing the examination of more complex and clustered mitochondrial structures.^94^

### Cryo-electron tomography (cryo-ET) liquid ethane vitrification

Mitochondria were freshly isolated from two healthy full-term placentae via sequential differential centrifugation.^53^ Briefly, 50 mg of villous placental tissue was hand-homogenised using a glass Dounce homogeniser, along with 1 mL of ice-cold mitochondrial isolation buffer (250 mM sucrose, 0.5 mM sodium EDTA, 10 mM tris).^53^ The non-mitochondrial cellular fraction was removed following centrifugation at 1500 x *g* for 10 min at 4 °C. The supernatant was then centrifuged at 4000 x *g* for 15 min at 4 °C, resulting in a Cyto-Mitochondria enriched pellet. The supernatant was centrifuged again at 12,000 × *g* for 15 min at 4 °C, resulting in a Syncytio-Mitochondria enriched pellet. Mitochondrial pellets were then resuspended in isolation buffer. From each placenta, three tissue biopsies were used to produce pooled and concentrated preparations enriched for Cyto- and Syncytio-Mitochondria.^53^ Further analysis was performed to examine the heterogeneity and purity of isolated Cyto- and Syncytio-Mitochondria, and revealed that both Cyto- and Syncytio-Mitochondria retain the expected transverse diameter of their in situ counterparts as observed via array tomography (Fig. 1), and are structurally divergent of villous endothelial or Hofbauer mitochondria in tissue (Supplementary Fig. 14). As soon as possible after birth, isolated mitochondrial samples were resuspended in 50 µL of isolation buffer and vitrified in liquid ethane using the Vitrobot Mark IV System (Thermo Fisher Scientific, USA). To do this, 3 µL of each sample was blotted for 5 s, with a blot force of 7, onto glow discharged Quantifoil R 1.2/1.3 300 mesh Au grids (Quantifoil, Germany). Glow discharging was performed using a Pelco EasiGlow (Pelco, USA) with a plasma current of 15 mA for 30 s, ultimate pressure set to 0.30 mbar, and stable pressure of 0.39 mbar.

### Cryo-ET image acquisition

Vitrified grids were clipped into AutoGrids, and imaged using the FEI Titan Krios 300 keV field emission gun TEM (Thermo Fisher Scientific, USA), equipped with a Gatan BioQuantum energy filter, and K3 camera system (AMETEK, Inc., USA). Single axis bidirectional tilt series were acquired using Tomography Software (Thermo Fisher Scientific, USA), with a range of ± 50°, at 3° increments and 3 s exposures for Cyto-Mitochondria, or 2° increments and 2 s exposures for Syncytio-Mitochondria. We identified that a higher exposure was necessary to improve the visualisation of inner membranes within the large and electron-dense Cyto-Mitochondria, whereas the smaller Syncytio-Mitochondria inner membranes were less obscured. The compromise, however, was that the incremental step size was also increased to limit the total electron dose and prevent beam-induced damage.^95^ Both sample types were imaged at 42,000× magnification, with a defocus range between −5 µm and −8 µm. Zero-loss imaging was performed with an energy filter width set to 15 eV, using the counted and super resolution modes on the K3 direct detector, and subsequently binned by 2 for reconstruction. The maximum accumulated electron dose for tomograms was 100 e^-^/Å^2^, with pixel sizes ranging between 2.1 Å and 3.4 Å.

### Cryo-ET tomogram reconstruction

Tilt series were processed using the IMOD software suite (University of Colorado, USA).^89^ Exposures with significant beam shift or obstructed views were excluded from the reconstruction process. Tilt series were aligned using fiducial-less patch tracking, prior to 2× binning to improve the signal to noise ratio. Tomograms were reconstructed using the back projection method, with a SIRT-like filter set to 20 iterations, using the Hamming-like filter.^96^

### Cryo-ET segmentation

Reconstructed tomograms were imported into AMIRA (Thermo Fisher Scientific, USA), and denoised using the median and non-local means filters to improve the contrast of mitochondrial membranes.^97,98^ Manual segmentation of Cyto-Mitochondria and Syncytio-Mitochondria (*n* = 8 mitochondria per cell type) was performed every 5–10 Z-slices and then interpolated—with interpolated segmentations checked for inaccuracies and significant deviations from the true membrane line. Slices with poor visualisation of deeper internal membranes were excluded from segmentation. Segmentations were meshed to produce 3D reconstructions of mitochondrial outer membranes and cristae. All 16 3D reconstructions are provided in the supplementary file (Supplementary Fig. 2).

### Quantification of intermembrane distance

For each mitochondrion, the tomographic slice with the best membrane visibility was chosen for all 2D measurements. Each mitochondrion was split into four Cartesian quadrants (Supplementary Fig. 3), and for each quadrant, the perpendicular distance between the outer and inner membrane was measured if it presented with clearly visible membranes (resulting in up to four intermembrane distance measurements per mitochondrion).

### Quantification of cristae length and width

The maximum XY length of each crista was measured, followed by the perpendicular maximum XY width. Elongation was calculated as a ratio between the length and width measurements (Supplementary Fig. 3).

### Quantification of cristae angle

For each lamellar cristae, the XY angles of junctions and apices were measured, while the cristae angle for tubulo-vesicular and globular cristae was measured using the quadrant system, with at least one angle measurement taken from each quadrant. Additional angles were measured for cristae with multiple deviations in membrane curvature (Supplementary Fig. 3).

### Quantification of surface area and volume

Cristae surface area was calculated using the membrane segmentations, whereas cristae volume was calculated using segmentations with filled cristae lumen. It was necessary to develop two distinct reconstructions to ensure that surface area measurements were not skewed by the flat ‘caps’ that were required to enclose the lumen of cristae for volume calculations. The volume of lamellar cristae was calculated by connecting the cristae junctions with a faux inner boundary membrane, thus enclosing the lumen, similar to a previously described workflow.^99^ Volume data was normalised to the number of tomographic slices for each mitochondrion.

### Quantification of 3D curvature

To characterise the complex topology and 3D curvature of cristae structure, six cristae reconstructions were first imported into MeshLab (ISTI-CNR, Italy), cleaned to remove flat “caps” that enclosed the cristae, and the Laplacian smoothing filter (set to 500 iterations) was applied to mitigate the interference of segmentation artefacts upon curvature estimation, while preserving large-scale features and gross topology.^100^ Smoothing was consistently applied to all reconstructions prior to the analysis of curvature, ensuring that segmentation edge effects were minimised. Smoothed reconstructions were then imported into ParaView (Sandia National Laboratories, USA), and principal curvatures were derived from mean and Gaussian curvature calculations using the Python calculator tool. Principal curvatures were subsequently used to calculate curvedness

3 $$C=\,\sqrt{\frac{{{k}_{1}}^{2}+\,{{k}_{2}}^{2}}{2}}$$

where C is curvedness, ${k}_{1}$ is maximum curvature, and ${k}_{2}$ is minimum curvature.^101^

As previously published, curvedness assesses the magnitude of curvature irrespective of direction, and is therefore more appropriate for comparing complex cristae topologies compared to mean and gaussian curvature measurements.^101^ The notable advantage of using ParaView to calculate curvedness, was the normalisation of colour maps, enabling a standardised comparison of multiple cristae reconstructions from different tomographic data.

### Statistical analysis of electron microscopy data

Array tomography and cryo-ET quantification data were processed using GraphPad Prism (GraphPad Software, USA), and first examined for outliers using Grubb’s Outliers test. The Shapiro-Wilk test was performed on all groups to assess the normality of distribution. To account for non-independence of data points, all array tomography data was examined using a linear mixed model analysis in SPSS (IBM, USA), with cell population as a fixed effect, and the placental sample as a random intercept to account for clustering. Cell population remained a significant predictor of variation between CTBs and the STB when accounting for placental-level clustering, which contributed towards 0–19% of variance, depending on the parameter. The statistical report is included in the supplementary file (Supplementary Figs. 4–9). The intermembrane distance in our cryo-ET reconstructions was examined using the Mann-Whitney U test. The Kruskal-Wallis and Dunn’s multiple comparisons tests (post-hoc) were applied to compare all cryo-ET metrics between lamellar, tubulo-vesicular, and globular cristae architectures, except for volume—which was normally distributed and therefore analysed using the Brown-Forsythe and Welch ANOVA test (post-hoc). Significant variation between groups was established as *p* < 0.05. Additional analysis shows array tomography data split by placental sample, which was analysed using the Kruskal-Wallis ANOVA with Dunn’s multiple comparisons test (post-hoc) for the purpose of observing significant variations between cell populations and placental samples (Supplementary Fig. 10).

### In silico analysis of proteomic data

This study performed an in silico analysis of previously acquired LC-MS data.^57^ Mitochondria were isolated from placental villous tissue of healthy full-term pregnancies as described above and in the referenced published protocol,^53^ and LC-MS was performed using enriched fractions of Cyto- and Syncytio-Mitochondria (*n* = 3 placentae).^57^ In contrast to the previous publication which only presented proteins deemed significant following Fisher’s exact test independent to fold change, this study performed an in silico analysis of the dataset to assess protein changes between mitochondrial populations within specific pathways. To determine the pathways, the following terms were used; NADH dehydrogenase [ubiquinone] (*n* = 24 subunits), succinate dehydrogenase [ubiquinone] (*n* = 1 subunit), cytochrome b-c1 (*n* = 6 subunits), cytochrome c oxidase (*n* = 5 subunits), ATP synthase (*n* = 10 subunits), in addition to select proteins that reside within mitochondrial dynamics (*n* = 4 proteins), or MIB complexes (*n* = 8 proteins). Proteins were normalised to Cyto-Mitochondria as previously described,^57^ and the Log_2_ fold change (Log_2_FC) identifies directionality and relative abundance between the populations. Proteins with less than three unique peptide sequences identified by LC-MS were excluded from analysis to improve the reliability and reproducibility of our results. Heatmap graphs were produced using GraphPad Prism (GraphPad Software, USA), with a positive Log_2_FC representing a greater abundance in Cyto-Mitochondria, and a negative Log_2_FC as a greater abundance in Syncytio-Mitochondria.

### Clear-native PAGE (CN-PAGE)

To perform CN-PAGE, Cyto- and Syncytio-Mitochondria isolates (n = 7 placentae) were resuspended in aminocaproic acid buffer (1.5 M aminocaproic acid, 50 mM Bis-Tris) and the protein concentration measured using a BCA assay kit. Digitonin was added to achieve a 1:1 protein/detergent ratio for each sample, and incubated for 10 min on ice.^102^ Following incubation, samples were centrifuged at 20,000 × *g* for 30 min at 4 °C and the pellet was discarded. To the supernatant, 1× native loading buffer (aminocaproic acid, 1 M Bis-Tris, 500 mM EDTA, Coomassie Brilliant blue G 250) was added, and 10 µL of each sample (5 µg of protein per well) was loaded into the wells of a 4–12% Bis-Tris gel (Invitrogen, USA). Protein size was assessed using 3 µL of NativeMark Unstained Protein Standard. Following this, the cathode and anode chambers of the electrophoresis tank were filled with 50 mM Bis-Tris. Electrophoresis was performed at 4 °C to prevent overheating, at a constant voltage of 300 V, until the lowest band of proteins reached the bottom of the gel. After washing the gel in 50 mM Bis-Tris for 30 min, imaging was performed using the Amersham Imager 600 (GE HealthCare, USA).

### Western blotting

Cyto- and Syncytio-Mitochondria isolates (*n* = 7 placentae) were resuspended in 250 µL of RIPA buffer (100 mM Tris, 300 mM sodium chloride, 10% Nonidet P40, 10% sodium deoxycholate, 1% sodium dodecyl sulfate, 5 x Complete Mini Protease Inhibitor Cocktail tablet (Roche Diagnostics, Australia), pH 7.4).^57^ Sample lysates were prepared to a normalised concentration of 40 µg/µL, with Milli-Q H_2_O, NuPAGE Sample Reducing Agent, and NuPAGE Loading Dye (Thermo Fisher Scientific, USA). Lysates were heated to 90 °C for 10 min, then stored at −80 °C until use. Electrophoresis was performed with chilled 1× NuPAGE MOPS SDS buffer (Invitrogen, USA), for 10 min at 100 V and then 90 min at 200 V. Transfer was performed using 1× transfer buffer (200 mL 10 x transfer buffer (72.5 g tris hydrochloride, 36.6 g glycine, 1 L H_2_O), 400 mL methanol, 1400 mL H_2_O), for 10 min at 100 V and 60 min at 200 V. Membranes were washed in 5 mL PBST wash buffer (100 mL 10× PBS, 2% Tween 20, 900 mL H_2_O) for 30 min at 4 °C, and then blocked for 1 h using 5 mL of Odyssey Blocking Buffer (LI-COR Biosciences, USA). Following a 15 min PBST wash, membranes were incubated with the following primary antibodies at a concentration of 1:250; anti-apolipoprotein O or MIC26 (ab246865, Abcam, UK) anti-SAMM50 (ab133709, Abcam, UK), and anti-ATP5MK (pa569671, Thermo Fisher Scientific, USA), or anti-beta actin at a concentration of 1:1000 (ab8226, Abcam, UK), overnight at 4 °C. Following a 30 min PBST wash, membranes were incubated in 1:20,000 IRDye 680LT goat anti-rabbit or IRDye 800CW goat anti-mouse sary antibody (LI-COR Biosciences, USA) for 1 hour. After a final 15 min PBST wash, membranes were imaged using the Odyssey M Imager (LI-COR Biosciences, USA). Full-size representative western blots are provided in the supplementary file (Supplementary Figs. 11–13), all other blots are available in the Source Data.

### Analysis of membrane blots

CN-PAGE and western blotting membrane images were analysed using Image Studio (LI-COR Biosciences, USA), and blot signal intensities were quantified relative to background noise. Supercomplexes were identified by molecular weight, as previously published.^103^ Western blots were run in experimental duplicates, with both membrane quantifications averaged prior to statistical analysis. GraphPad Prism (GraphPad Software, USA) was used to perform Grubb’s Outliers Test, followed by the Mann-Whitney U test to assess ATP5MK, and unpaired t-tests for all other proteins. Significant variation was established as *p* < 0.05.

### Reporting summary

Further information on research design is available in the Nature Portfolio Reporting Summary linked to this article.

## Supplementary information

Supplementary Information

Reporting Summary

Transparent Peer Review file

## Source data

Source Data

## Data Availability

The array tomography data generated in this study have been deposited in the European Bioinformatics Institute (EBI) BioImage Archive under accession code S-BIAD3408. The cryo-ET tilt series and reconstructed tomograms are available on EBI BioImage Archive under accession code S-BIAD3409. This study performed an in silico analysis of a legacy proteomics dataset; therefore, LC-MS data is included in the Source Data file, and.RAW LC-MS acquisition files are available upon reasonable request. Source data are provided with this paper.
