# Supplementary material for: Electron tomography reveals mitochondrial network and cristae remodelling during cell differentiation in the human placenta
Source: Nat Commun. 2026 Jul 8;17:8407. doi: 10.1038/s41467-026-75408-8 (PMC13473558; doi:10.1038/s41467-026-75408-8)
Supplement: Supplementary file 1 [file 41467_2026_75408_MOESM1_ESM.pdf]

Electron tomography reveals mitochondrial network and cristae remodelling during cell differentiation in the human placenta

Authors

Siddharth Acharya, Eric Hanssen, Veronica B. Botha, Tia M. Smith, Sahan Jayatissa, Zlatan Trifunovic, Lucy A. Bartho, John E. Schjenken, Tu'uhevaha J. Kaitu'u-Lino, Anthony V. Perkins, Joanna L. James, Kirsty G. Pringle, James C. Bouwer, Roger Smith, Joshua J. Fisher

Supplementary File

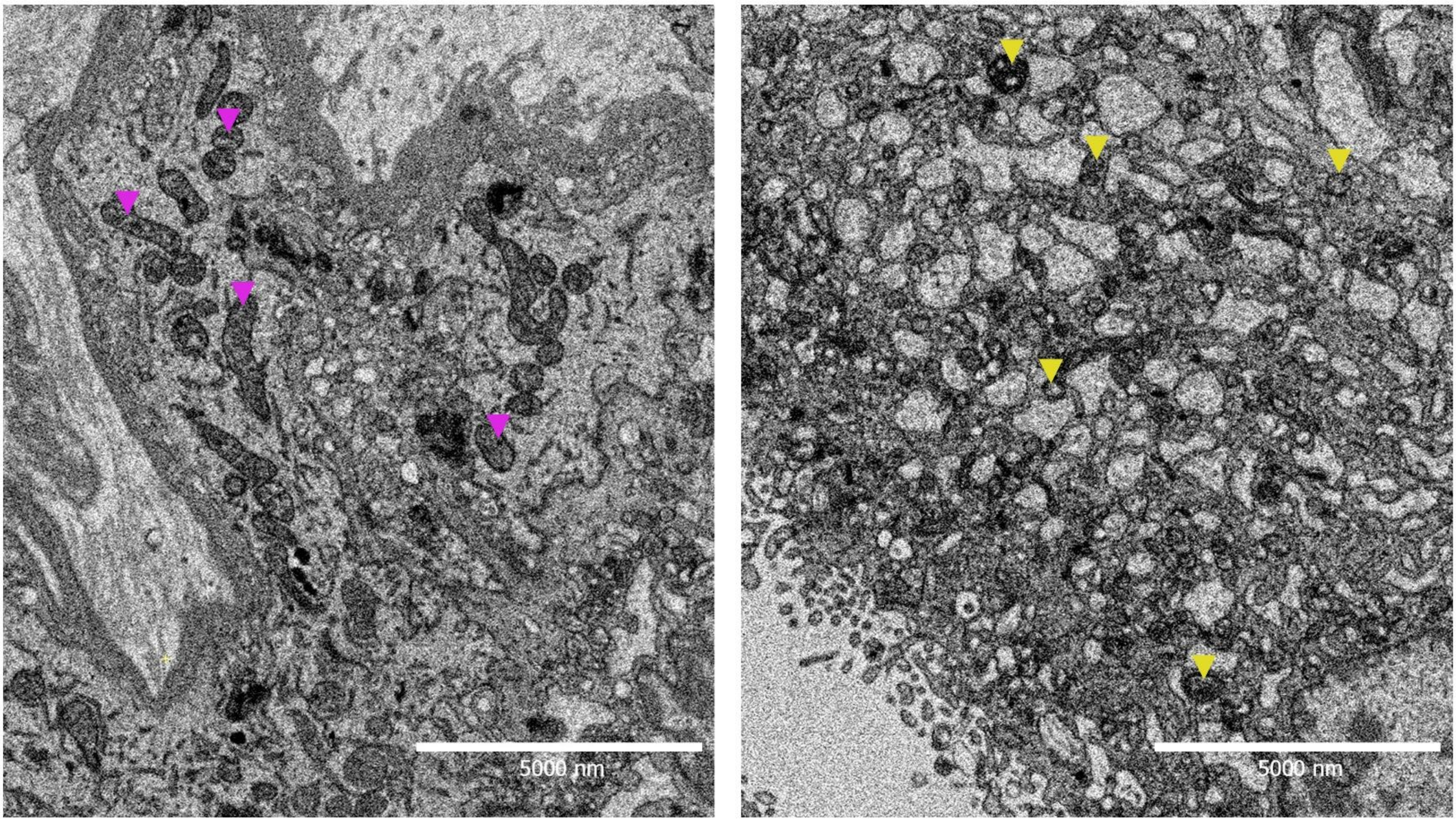

Supplementary Figure 1: Array tomography of placental tissue, representative mitochondria from CTB and STB. Left: CTB cell with Cyto-Mitochondria indicated by purple arrowheads. Cyto-Mitochondria are enclosed by a double-membrane that appears electron-dense and dark, with internal cristae membranes and a dense, grey matrix. Right: STB with small, electron-dense Syncytio-Mitochondria indicated by yellow arrowheads. Syncytio-Mitochondria are small, punctate, and extremely electron-dense.

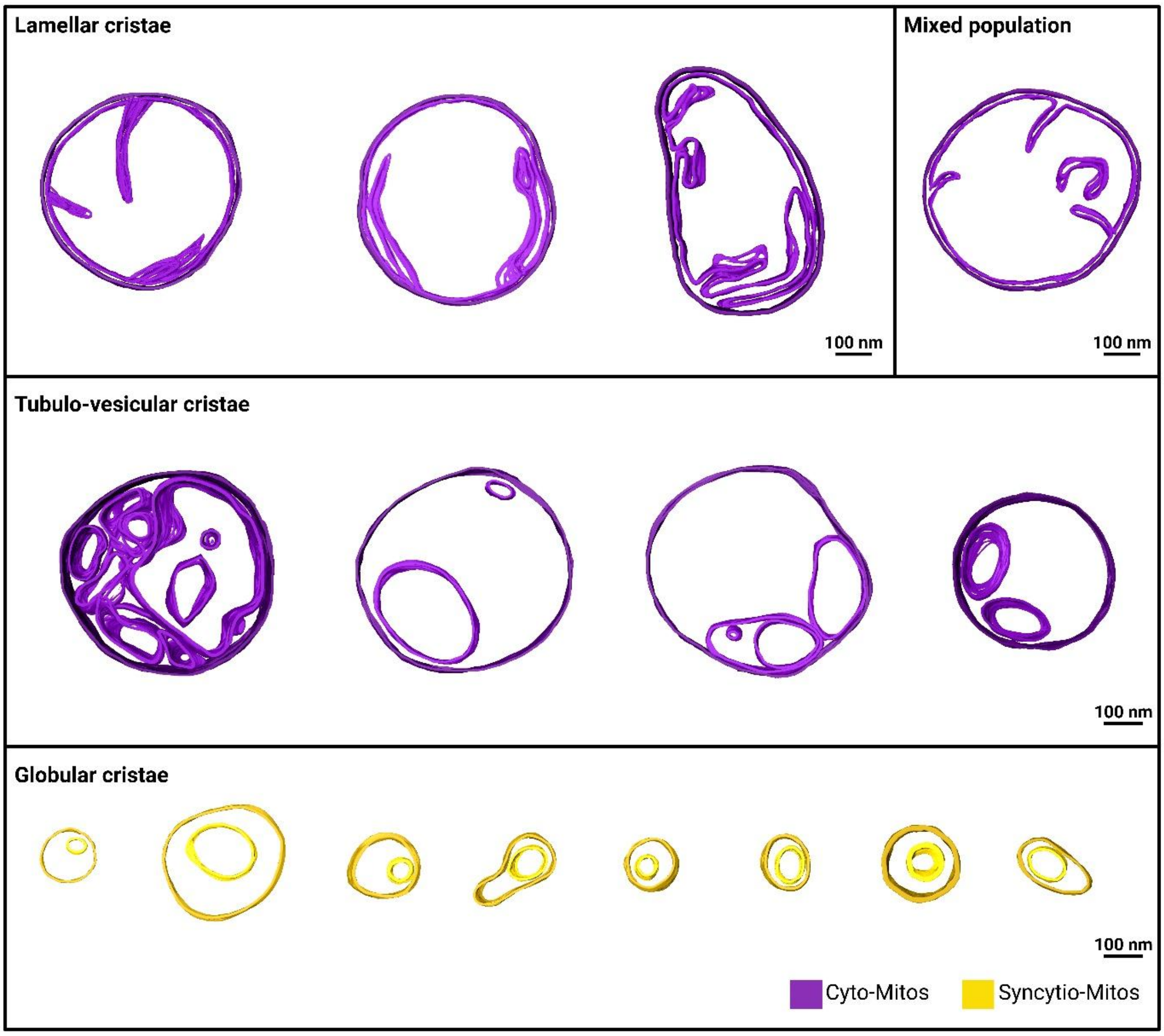

Supplementary Figure 2: Overview of all mitochondria analysed by cryo-ET. 3D reconstructions were produced from tomographic data of Cyto- and Syncytio-Mitochonria. Two primary mitochondrial subpopulations are observed in the CTB (purple), consisting of lamellar cristae or tubulo-vesicular cristae, in addition to what may be a third subpopulation consisting of a combination of both cristae architectures "mixed population". A homogeneous mitochondrial subpopulation is observed in the STB (yellow), of globular cristae – with the exception of one mitochondrion that consisted of irregular shaped cristae (dashed box). Scale bar represents 100 nm.

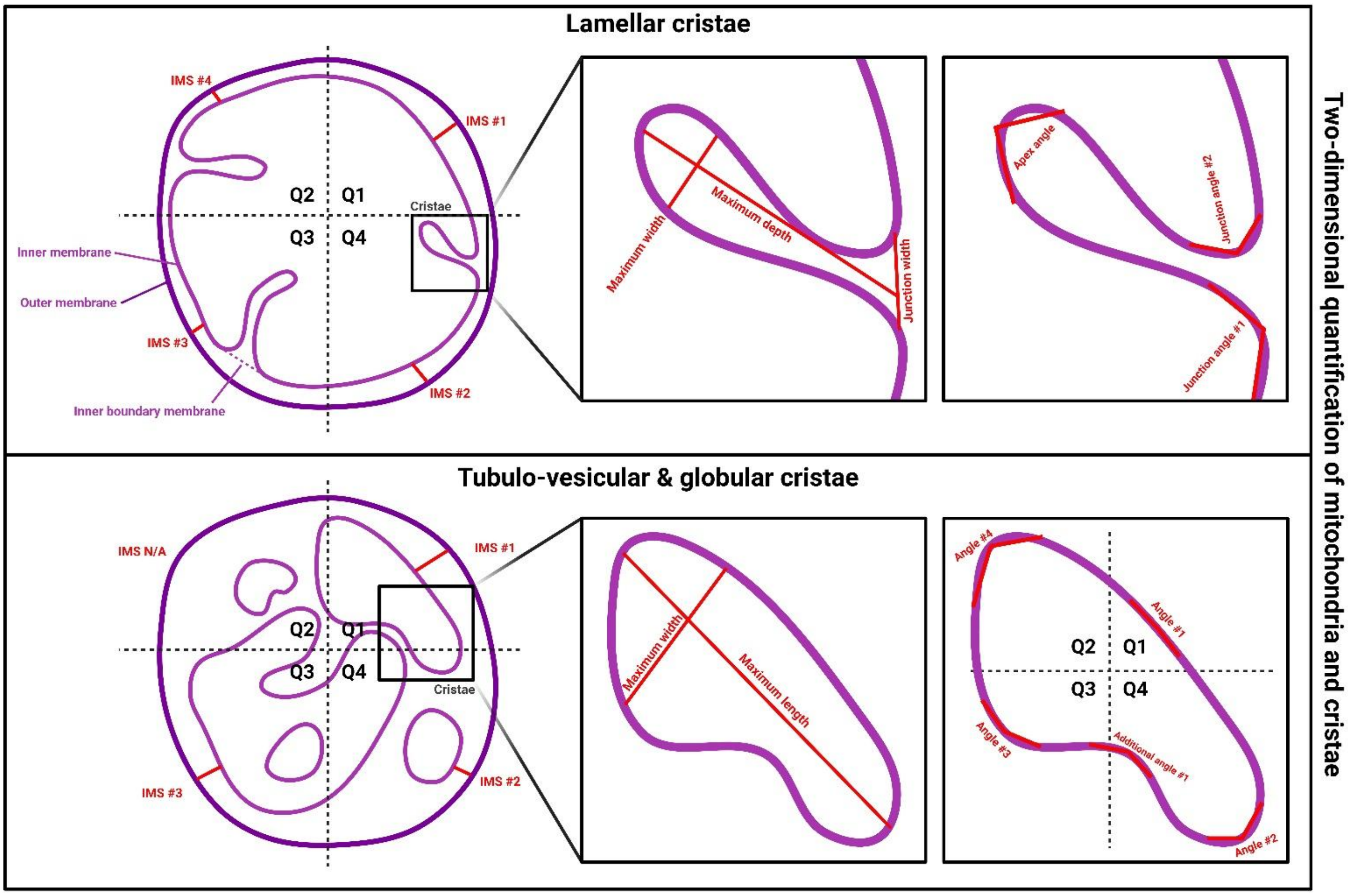

Supplementary Figure 3: Quantification of 2D mitochondrial and cristae structure. Representative diagram showing the method for acquiring 2D metrics for lamellar cristae (above) or tubulo-vesicular/globular cristae (below). The tomographic slice with the best presentation of clear membranes was chosen for performing measurements. Each mitochondrion was split into four cartesian quadrants, and an intermembrane space measurement was made in each quadrant if there were clear parallel outer and inner membranes. For each lamellar cristae, the maximum depth and perpendicular maximum width was measured. Each apex and opposing junction angles were measured for all lamellar cristae. For tubulo-vesicular and globular cristae, maximum length and perpendicular maximum width was measured, and the cristae cross-section was split into four cartesian quadrants to measure the membrane curvature in each quadrant. In addition, angle measurements were recorded for any significant deviatoric curvatures (as shown by the representative “additional angle #1”).

Type III Tests of Fixed Effects[a]

Source	Numerator df	Denominator df	F	Sig.
Intercept	1	.993	25.301	.126
Cell	1	328.377	154.150	<.001

a. Dependent Variable: Surface_area.

Intraclass Correlation Coefficients

Overall ICCs	Adjusted	.187
	Conditional	.128

Estimates of Fixed Effects[a]

Parameter	Estimate	Std. Error	df	t	Sig.	95% Confidence Interval	
						Lower Bound	Upper Bound
Intercept	.596	.219	1.029	2.717	.219	-2.010	3.201
[Cell=1.00]	.994	.080	328.377	12.416	<.001	.836	1.151
[Cell=2.00]	0[b]	0	.	.	.	.	.

a. Dependent Variable: Surface_area.

b. This parameter is set to zero because it is redundant.

Estimates of Covariance Parameters[a]

Parameter		Estimate	Std. Error	Wald Z	Sig.	95% ...
						Lower Bound
Residual		.399	.031	12.806	<.001	.342
Intercept [subject = Placenta]	Variance	.092	.134	.684	.494	.005

Supplementary Figure 4: Linear mixed model analysis of array tomography data (surface area). A linear mixed model analysis was performed to examine statistical variation between CTB and STB mitochondrial surface area, with cell population as a fixed effect, and placental sample as a random intercept to account for clustering. Included are the results from Type III tests of fixed effects, the intraclass correlation coefficient, estimates of fixed effects, and estimates of covariance parameters. Analysis was performed in SPSS (IBM, USA).

Type III Tests of Fixed Effects[a]

Source	Numerator df	Denominator df	F	Sig.
Intercept	1	.999	18.777	.145
Cell	1	344.877	170.736	<.001

a. Dependent Variable: Volume.

Intraclass Correlation Coefficients

Overall ICCs	Adjusted	.167
	Conditional	.114

Estimates of Fixed Effects[a]

Parameter	Estimate	Std. Error	df	t	Sig.	95% Confidence Interval	
						Lower Bound	Upper Bound
Intercept	.034	.019	1.028	1.844	.311	-.187	.255
[Cell=1.00]	.091	.007	344.877	13.067	<.001	.077	.105
[Cell=2.00]	0[b]	0	.	.	.	.	.

a. Dependent Variable: Volume.

b. This parameter is set to zero because it is redundant.

Estimates of Covariance Parameters[a]

Parameter		Estimate	Std. Error	Wald Z	Sig.	95% ...
						Lower Bound
Residual		.003	.000	13.115	<.001	.003
Intercept [subject = Placenta]	Variance	.001	.001	.684	.494	3.736e-5

Supplementary Figure 5: Linear mixed model analysis of array tomography data (volume). A linear mixed model analysis was performed to examine statistical variation between CTB and STB mitochondrial volume, with cell population as a fixed effect, and placental sample as a random intercept to account for clustering. Included are the results from Type III tests of fixed effects, the intraclass correlation coefficient, estimates of fixed effects, and estimates of covariance parameters. Analysis was performed in SPSS (IBM, USA).

Type III Tests of Fixed Effects[a]

Source	Numerator df	Denominator df	F	Sig.
Intercept	1	.963	1990.542	.016
Cell	1	87.959	134.661	<.001

a. Dependent Variable: SA_V.

Intraclass Correlation Coefficients

Overall ICCs	Adjusted	.003
	Conditional	.002

Estimates of Fixed Effects[a]

Parameter	Estimate	Std. Error	df	t	Sig.	95% Confidence Interval Lower Bound	95% Confidence Interval Upper Bound
Intercept	22.658	.464	1.285	48.787	.005	19.106	26.210
[Cell=1.00]	-8.234	.710	87.959	-11.604	<.001	-9.644	-6.824
[Cell=2.00]	0[b]	0	.	.	.	.	.

a. Dependent Variable: SA_V.

b. This parameter is set to zero because it is redundant.

Estimates of Covariance Parameters[a]

Parameter		Estimate	Std. Error	Wald Z	Sig.	95% ... Lower Bound
Residual		37.122	2.822	13.153	<.001	31.983
Intercept [subject = Placenta]	Variance	.104	.498	.208	.835	8.330e-6

Supplementary Figure 6: Linear mixed model analysis of array tomography data (SA:V). A linear mixed model analysis was performed to examine statistical variation between CTB and STB mitochondrial SA:V, with cell population as a fixed effect, and placental sample as a random intercept to account for clustering. Included are the results from Type III tests of fixed effects, the intraclass correlation coefficient, estimates of fixed effects, and estimates of covariance parameters. Analysis was performed in SPSS (IBM, USA).

Type III Tests of Fixed Effects[a]

Source	Numerator df	Denominator df	F	Sig.
Intercept	1	.990	377.432	.034
Cell	1	308.050	62.305	<.001

a. Dependent Variable: XY_elongation.

Intraclass Correlation Coefficients

Overall ICCs	Adjusted	.032
	Conditional	.027

Estimates of Fixed Effects[a]

Parameter	Estimate	Std. Error	df	t	Sig.	95% Confidence Interval	
						Lower Bound	Upper Bound
Intercept	1.280	.083	1.132	15.458	.030	.477	2.082
[Cell=1.00]	.544	.069	308.050	7.893	<.001	.408	.680
[Cell=2.00]	0[b]	0	.	.	.	.	.

a. Dependent Variable: XY_elongation.

b. This parameter is set to zero because it is redundant.

Estimates of Covariance Parameters[a]

Parameter		Estimate	Std. Error	Wald Z	Sig.	95% ...
						Lower Bound
Residual		.324	.025	13.115	<.001	.279
Intercept [subject = Placenta]	Variance	.011	.018	.586	.558	.000

Supplementary Figure 7: Linear mixed model analysis of array tomography data (XY elongation). A linear mixed model analysis was performed to examine statistical variation between CTB and STB mitochondrial XY elongation, with cell population as a fixed effect, and placental sample as a random intercept to account for clustering. Included are the results from Type III tests of fixed effects, the intraclass correlation coefficient, estimates of fixed effects, and estimates of covariance parameters. Analysis was performed in SPSS (IBM, USA).

Type III Tests of Fixed Effects[a]

Source	Numerator df	Denominator df	F	Sig.
Intercept	1	1.000	1809.737	.015
Cell	1	340.481	20.671	<.001

a. Dependent Variable: Sphericity.

Intraclass Correlation Coefficients

Overall ICCs	Adjusted	.069
	Conditional	.065

Estimates of Fixed Effects[a]

Parameter	Estimate	Std. Error	df	t	Sig.	95% Confidence Interval	
						Lower Bound	Upper Bound
Intercept	.816	.019	1.073	43.120	.011	.611	1.021
[Cell=1.00]	-.051	.011	340.481	-4.547	<.001	-.073	-.029
[Cell=2.00]	0[b]	0	.	.	.	.	.

a. Dependent Variable: Sphericity.

b. This parameter is set to zero because it is redundant.

Estimates of Covariance Parameters[a]

Parameter		Estimate	Std. Error	Wald Z	Sig.	95% ...
						Lower Bound
Residual		.009	.001	13.153	<.001	.007
Intercept [subject = Placenta]	Variance	.001	.001	.650	.516	3.109e-5

Supplementary Figure 8: Linear mixed model analysis of array tomography data (sphericity). A linear mixed model analysis was performed to examine statistical variation between CTB and STB mitochondrial sphericity, with cell population as a fixed effect, and placental sample as a random intercept to account for clustering. Included are the results from Type III tests of fixed effects, the intraclass correlation coefficient, estimates of fixed effects, and estimates of covariance parameters. Analysis was performed in SPSS (IBM, USA).

Type III Tests of Fixed Effects[a]

Source	Numerator df	Denominator df	F	Sig.
Intercept	1	.996	120.367	.058
Cell	1	339.305	21.879	<.001

a. Dependent Variable: MCI.

Intraclass Correlation Coefficients

Overall ICCs	Adjusted	.074
	Conditional	.070

Estimates of Fixed Effects[a]

Parameter	Estimate	Std. Error	df	t	Sig.	95% ... Lower Bound
Intercept	1.428	.151	1.063	9.441	.059	-.243
[Cell=1.000000]	.407	.087	339.305	4.678	<.001	.236
[Cell=2.000000]	0[b]	0	.	.	.	.

Estimates of Covariance Parameters[a]

Parameter		Estimate	Std. Error	Wald Z	Sig.	95% ... Lower Bound
Residual		.510	.039	13.115	<.001	.439
Intercept [subject = Placenta]	Variance	.041	.063	.652	.514	.002

Supplementary Figure 9: Linear mixed model analysis of array tomography data (mitochondrial complexity index; MCI). A linear mixed model analysis was performed to examine statistical variation between CTB and STB mitochondrial MCI, with cell population as a fixed effect, and placental sample as a random intercept to account for clustering. Included are the results from Type III tests of fixed effects, the intraclass correlation coefficient, estimates of fixed effects, and estimates of covariance parameters. Analysis was performed in SPSS (IBM, USA).

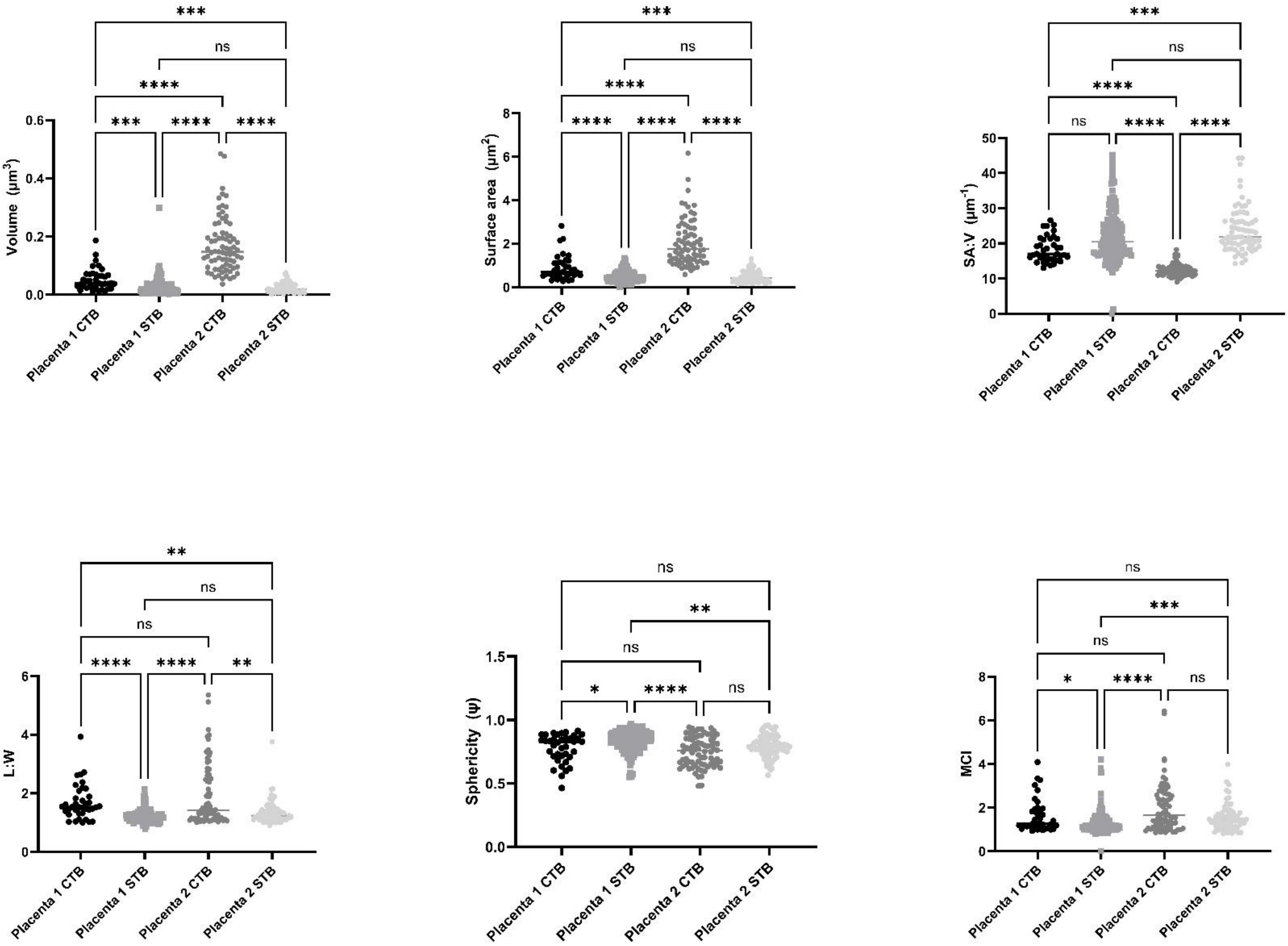

Supplementary Figure 10: Array tomography volumetric data split by patient. Volumetric analysis was performed on randomly selected subsets of mitochondria obtained from two CTBs and two STBs, collectively obtained from two healthy full-term placentae. Each placenta's mitochondrial volumetric data is shown to assess the inter-patient variability. Placenta 1 CTB n = 38; placenta 2 CTB n = 75; placenta 1 STB n = 170; placenta 2 STB n = 70. Kruskal-Wallis ANOVA with Dunn's multiple comparisons test was performed to assess significant variation between all groups, expressed as $p < 0.05$*, $p \leq 0.01$**, $p \leq 0.001$***, $p \leq 0.0001$****.

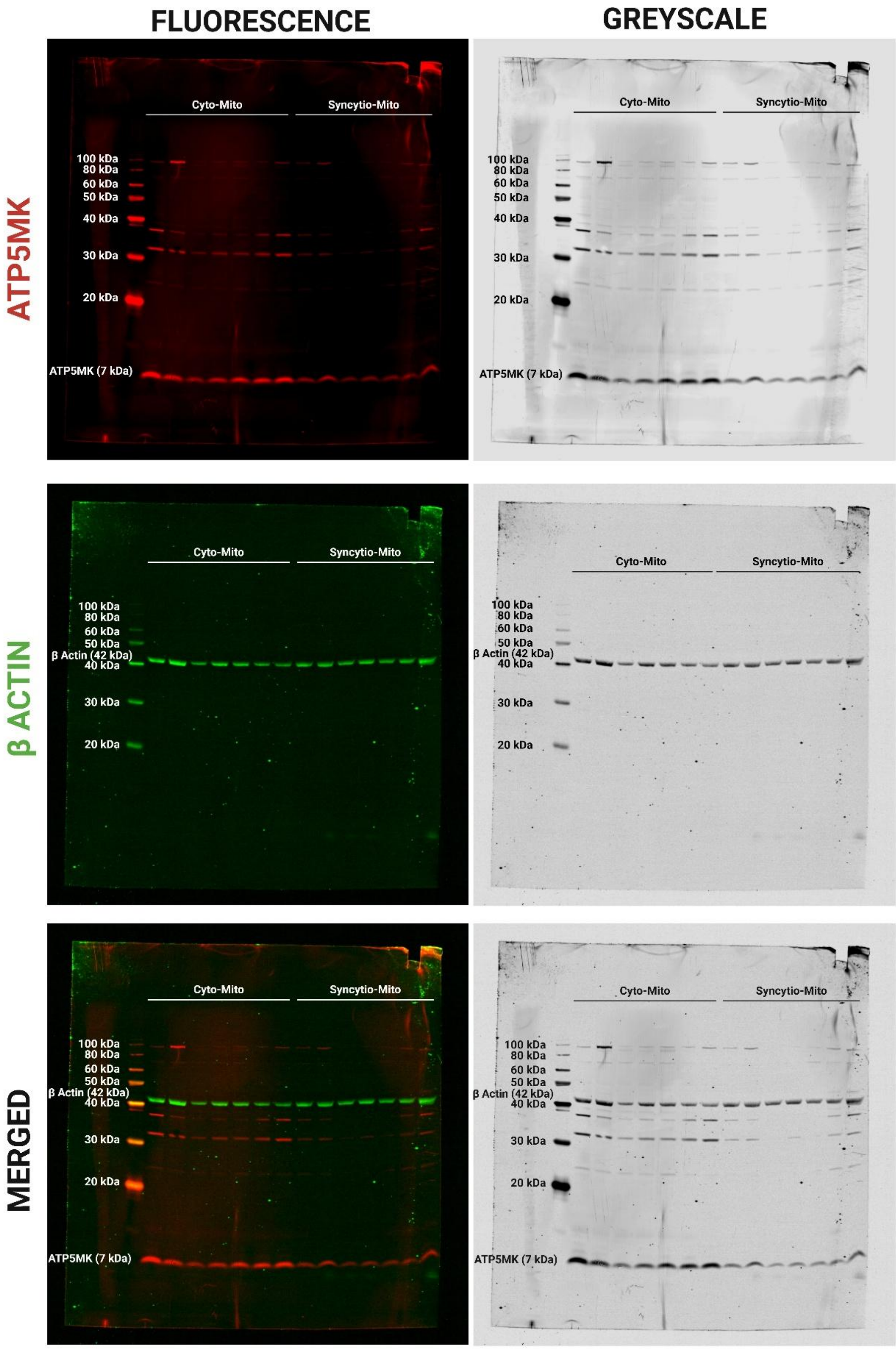

Supplementary Figure 11: Western blot of ATP5MK and β Actin. Western blotting was performed using mitochondrial isolates, derived from seven healthy full-term placentae. Top: ATP5MK (7 kDa) imaged using the red channel, middle: β Actin (42 kDa) imaged using the green channel, bottom: merged channels showing the relative position of the target protein of interest with β Actin. All images are shown in fluorescence (left) and grayscale (right). Lane 1: ladder, lane 2-8: Cyto-Mitochondria, lane 9-15: Syncytio-Mitochondria.

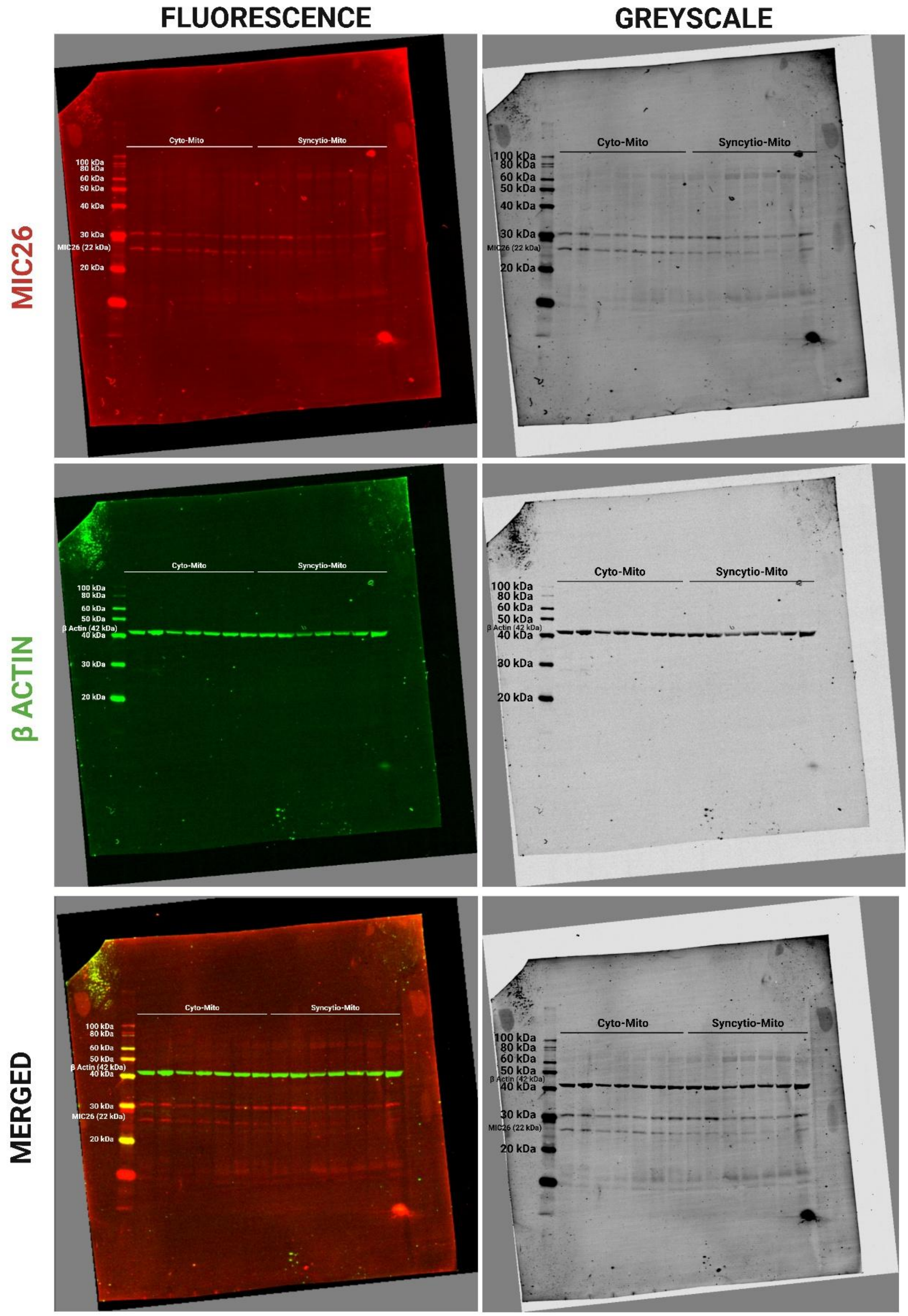

Supplementary Figure 12: Western blot of MIC26 and β Actin. Western blotting was performed using mitochondrial isolates, derived from seven healthy full-term placentae. Top: MIC26 (22 kDa) imaged using the red channel, middle: β Actin (42 kDa) imaged using the green channel, bottom: merged channels showing the relative position of the target protein of interest with β Actin. All images are shown in fluorescence (left) and grayscale (right). Lane 1: ladder, lane 2-8: Cyto-Mitochondria, lane 9-15: Syncytio-Mitochondria.

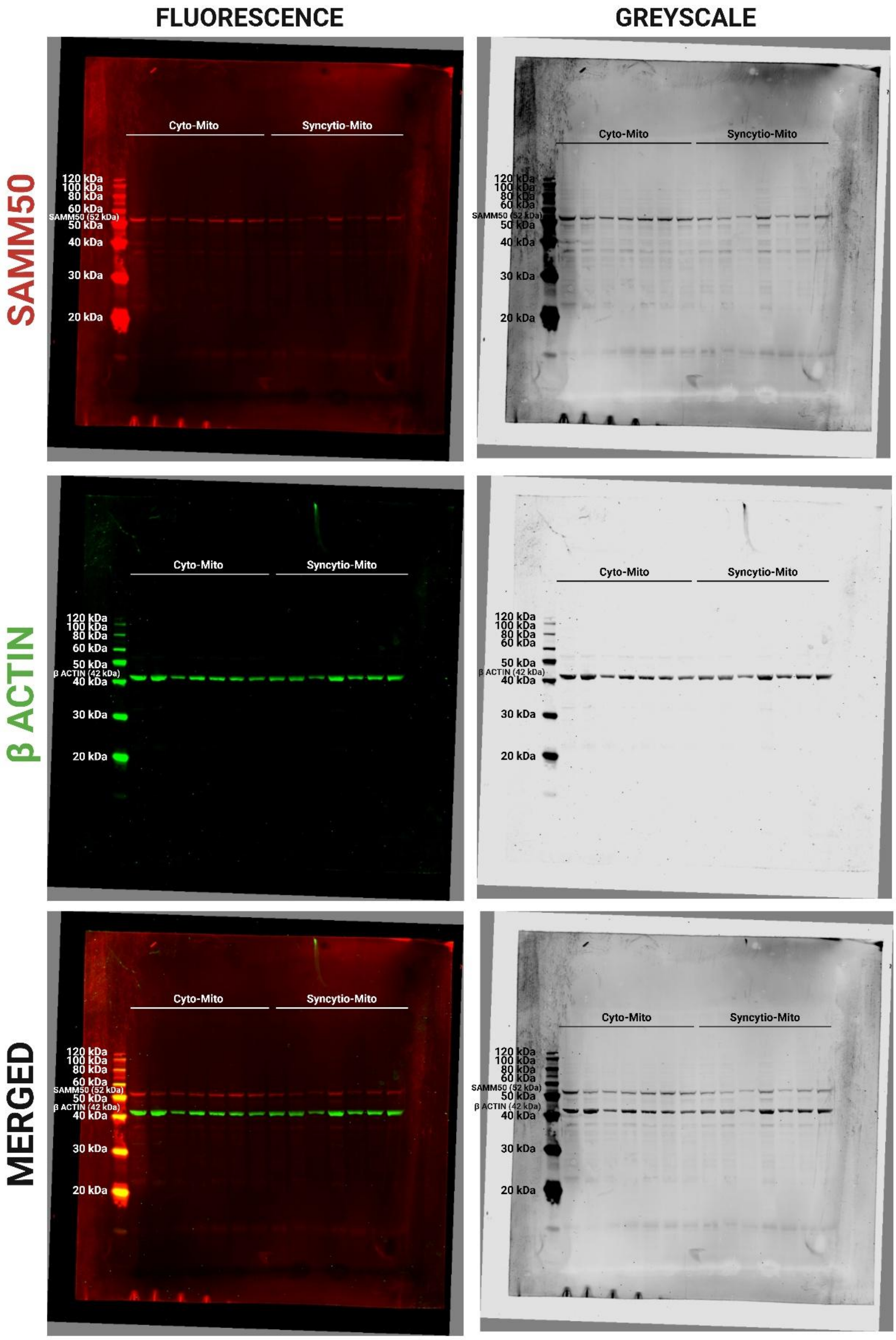

Supplementary Figure 13: Western blot of SAMM50 and β Actin. Western blotting was performed using mitochondrial isolates, derived from seven healthy full-term placentae. Top: SAMM50 (52 kDa) imaged using the red channel, middle: β Actin (42 kDa) imaged using the green channel, bottom: merged channels showing the relative position of the target protein of interest with β Actin. All images are shown in fluorescence (left) and grayscale (right). Lane 1: ladder, lane 2-8: Cyto-Mitochondria, lane 9-15: Syncytio-Mitochondria.

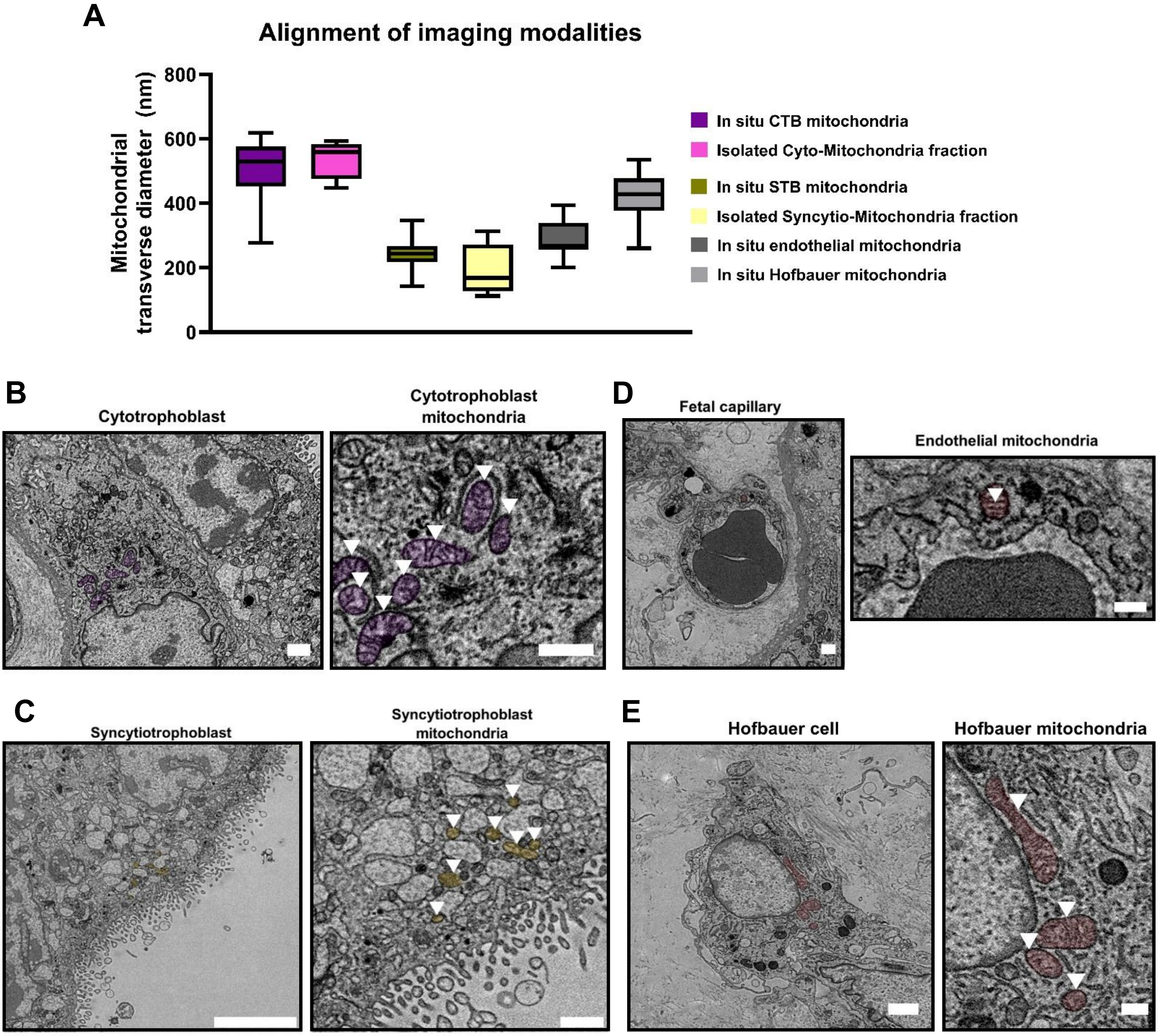

Supplementary Figure 14: Persistence of distinct mitochondrial phenotype following isolation. A) The graph demonstrates that isolated Cyto- and Syncytio-Mitochondria (cryo-ET) retain the expected diameter distributions of their in situ counterparts (array tomography). Interquartile ranges do not overlap across groups, and clear separation of central distributions between endothelial, Hofbauer, CTB, and STB mitochondria indicates the absence of structurally divergent and contaminating mitochondria within the mitochondrial isolations. In situ CTB mitochondria (n=35; purple), Isolated Cyto-Mitochondria fraction (n=8; pink), In situ STB mitochondria (n=35; dark yellow), Isolated Syncytio-Mitochondria fraction (n=8; light yellow), In situ endothelial mitochondria (n=20; dark grey), In situ Hofbauer mitochondria (n=20; light grey). Micrographs below the graph showcase a B) CTB cell with a magnified region of CTB mitochondria with variably orientated lamellar and tubulo-vesicular cristae (scale bars represent 1 μm); C) STB (scale bar represents 500 nm), with a magnified region of STB mitochondria (scale bar represents 100 nm); D) fetal capillary (scale bar represents 1 μm), with a magnified region of endothelial mitochondria with packed, aligned lamellar cristae (scale bar represents 500 nm); and E) Hofbauer cell (scale bar

represents 2 μm), with a magnified region of Hofbauer mitochondria with packed, aligned lamellar cristae (scale bar represents 500 nm).
