# Supplementary material for: Electron tomography reveals mitochondrial network and cristae remodelling during cell differentiation in the human placenta
Source: Nat Commun. 2026 Jul 8;17:8407. doi: 10.1038/s41467-026-75408-8 (PMC13473558; doi:10.1038/s41467-026-75408-8)
Supplement: Supplementary file 2 [file 41467_2026_75408_MOESM2_ESM.pdf]

nature portfolio

Corresponding author(s): Joshua J. Fisher

Last updated by author(s): Feb 27, 2026

Reporting Summary

Nature Portfolio wishes to improve the reproducibility of the work that we publish. This form provides structure for consistency and transparency in reporting. For further information on Nature Portfolio policies, see our Editorial Policies and the Editorial Policy Checklist.

Statistics

For all statistical analyses, confirm that the following items are present in the figure legend, table legend, main text, or Methods section.

n/a	Confirmed	
☐	☒	The exact sample size (*n*) for each experimental group/condition, given as a discrete number and unit of measurement
☐	☒	A statement on whether measurements were taken from distinct samples or whether the same sample was measured repeatedly
☐	☒	The statistical test(s) used AND whether they are one- or two-sided<br>*Only common tests should be described solely by name; describe more complex techniques in the Methods section.*
☐	☒	A description of all covariates tested
☐	☒	A description of any assumptions or corrections, such as tests of normality and adjustment for multiple comparisons
☐	☒	A full description of the statistical parameters including central tendency (e.g. means) or other basic estimates (e.g. regression coefficient) AND variation (e.g. standard deviation) or associated estimates of uncertainty (e.g. confidence intervals)
☒	☐	For null hypothesis testing, the test statistic (e.g. *F*, *t*, *r*) with confidence intervals, effect sizes, degrees of freedom and *P* value noted<br>*Give P values as exact values whenever suitable.*
☒	☐	For Bayesian analysis, information on the choice of priors and Markov chain Monte Carlo settings
☐	☒	For hierarchical and complex designs, identification of the appropriate level for tests and full reporting of outcomes
☒	☐	Estimates of effect sizes (e.g. Cohen's *d*, Pearson's *r*), indicating how they were calculated

Our web collection on statistics for biologists contains articles on many of the points above.

Software and code

Policy information about availability of computer code

Data collection	The following commercial software was used in data collection: MAPS (Thermo Fisher Scientific), Tomography 5 (Thermo Fisher Scientific).
Data analysis	The following commercial software was used in the analysis of data: AMIRA (Thermo Fisher Scientific), Graphpad Prism (Graphpad Software), Image Studio (LI-COR Biosciences), SPSS (IBM). The following open-source software was used: IMOD (University of Colorado, Boulder), ParaView (Sandia National Laboratories).

For manuscripts utilizing custom algorithms or software that are central to the research but not yet described in published literature, software must be made available to editors and reviewers. We strongly encourage code deposition in a community repository (e.g. GitHub). See the Nature Portfolio guidelines for submitting code & software for further information.

Data

Policy information about availability of data

All manuscripts must include a data availability statement. This statement should provide the following information, where applicable:

- Accession codes, unique identifiers, or web links for publicly available datasets
- A description of any restrictions on data availability
- For clinical datasets or third party data, please ensure that the statement adheres to our policy

The array tomography data generated in this study have been deposited in the European Bioinformatics Institute (EBI) BioImage Archive under accession code S-BIAD3408 [https://www.ebi.ac.uk/biostudies/bioimages/studies/S-BIAD3408]. The cryo-ET tilt series and reconstructed tomograms are available on EBI BioImage Archive under accession code S-BIAD3409 [https://www.ebi.ac.uk/biostudies/bioimages/studies/S-BIAD3409]. This study performed an in silico analysis of a legacy

proteomics dataset, therefore LC-MS data is included in the Source Data file, and .RAW LC-MS acquisition files are available upon reasonable request.

Research involving human participants, their data, or biological material

Policy information about studies with human participants or human data. See also policy information about sex, gender (identity/presentation), and sexual orientation and race, ethnicity and racism.

Reporting on sex and gender	This study does not discuss data based on sex or gender. Given the fundamental biology assessed in this study, the sample size is insufficient to ascertain sex and gender specific differences.
Reporting on race, ethnicity, or other socially relevant groupings	This study does not discuss data based on race, ethnicity, or other socially relevant groupings. Given the fundamental biology assessed in this study, the sample size is insufficient to ascertain race, ethnicity, or other socially relevant grouping specific differences.
Population characteristics	This study does not discuss, interpret or infer any data relevant to population characteristics.
Recruitment	With informed consent, placental samples were collected from healthy full-term pregnancies (38-40 weeks) at the John Hunter Hospital (NSW, Australia) and Mercy Hospital for Women (VIC, Australia).
Ethics oversight	Ethical approval was granted by the University of Newcastle Research Ethics Committee (H-382-0602), Hunter New England Health Human Research Ethics Committee (02/06/12/.13), Mercy Health Human Research Ethics Committee (R11/34), Griffith University Human Research Ethics Committee (MSC/05/15HREC), Queensland Government Human Research Ethics Committee (HREC/14/QPCH/246), and Site Specific Assessment (SSA/15/HNE/291) in compliance with the Declaration of Helsinki standards.

Note that full information on the approval of the study protocol must also be provided in the manuscript.

Field-specific reporting

Please select the one below that is the best fit for your research. If you are not sure, read the appropriate sections before making your selection.

[x] Life sciences [ ] Behavioural & social sciences [ ] Ecological, evolutionary & environmental sciences

For a reference copy of the document with all sections, see nature.com/documents/nr-reporting-summary-flat.pdf

Life sciences study design

All studies must disclose on these points even when the disclosure is negative.

Sample size	Experimental replicates (n=2) refers to the fixed factor, placental sample, in the linear mixed model. Specific focus was chosen on cell type (n=2), and the mitochondria therein (n=113 for cell 1, n=241 for cell 2). The use of multiple placentas and numerous cells allowed for a diverse, heterogeneous mitochondrial population in which to assess differences in structure, pre- and post-cellular differentiation. Cryo-electron tomography was performed on isolated mitochondria (n=16) derived from an additional two placentae. Proteomic analysis was performed (n=3 for each cellular mitochondrial subpopulation) and validated using western blotting and clear native PAGE in an additional seven placentae separated into the two cell types. Collectively, the findings of this study are derived from n=14 placentae, providing significant power for statistical analysis.
Data exclusions	Outliers were assessed using the Grubb's Outliers test, set to a standard alpha value of 0.05.
Replication	To ensure measures were reproducible, multiple placentae were sampled, multiple cells were chosen, and multiple mitochondria were analysed to identify the morphological changes in mitochondria between cells (array tomography). An additional n=2 placentae were used to confirm the structural changes via cryo-electron tomography. Mechanisms for structural changes were discovered in an additional n=3 placentae (proteomics), and subsequently validated by alternative methods in an additional n=7 placentae (western blotting and clear-native PAGE).<br>Replication and validation of our findings consistently across multiple independent methods and samples, greatly improve the reproducibility of this study.
Randomization	The mitochondrial 'subset' in this study refers to randomly selected mitochondria within our array tomography data as discussed extensively in the methods and supplementary material.
Blinding	Blinding was not relevant or required for the analysis of this data.

Reporting for specific materials, systems and methods

We require information from authors about some types of materials, experimental systems and methods used in many studies. Here, indicate whether each material, system or method listed is relevant to your study. If you are not sure if a list item applies to your research, read the appropriate section before selecting a response.

Materials & experimental systems

n/a	Involved in the study
[ ]	[x] Antibodies
[x]	[ ] Eukaryotic cell lines
[x]	[ ] Palaeontology and archaeology
[x]	[ ] Animals and other organisms
[x]	[ ] Clinical data
[x]	[ ] Dual use research of concern
[x]	[ ] Plants

Methods

n/a	Involved in the study
[x]	[ ] ChIP-seq
[x]	[ ] Flow cytometry
[x]	[ ] MRI-based neuroimaging

Antibodies

Antibodies used	The following antibodies were used in this study and all relevant information is included in text: anti-apolipoprotein O or MIC26 (ab246865, Abcam, UK) anti-SAMM50 (ab133709, Abcam, UK), anti-beta actin (ab8226, Abcam, UK), and anti-ATP5MK (pa569671, Thermo Fisher Scientific, USA), IRDye 680LT goat anti-rabbit, and IRDye 800CW goat anti-mouse secondary antibody (LI-COR Biosciences, USA).
Validation	Validation was performed by the antibody supplier, and the concentration was optimised prior to this study.

Plants

Seed stocks	N/A
Novel plant genotypes	N/A
Authentication	N/A
