# Supplementary material for: Electron tomography reveals mitochondrial network and cristae remodelling during cell differentiation in the human placenta
Source: Nat Commun. 2026 Jul 8;17:8407. doi: 10.1038/s41467-026-75408-8 (PMC13473558; doi:10.1038/s41467-026-75408-8)
Supplement: Supplementary file 3 [file 41467_2026_75408_MOESM3_ESM.pdf]

nature portfolio

Peer Review File

Electron tomography reveals mitochondrial network and cristae remodelling during cell differentiation in the human placenta

Corresponding Author: Dr Joshua Fisher

This file contains all reviewer reports in order by version, followed by all author rebuttals in order by version.

Version 0:

Reviewer comments:

Reviewer #1

(Remarks to the Author)
Summary: In this study, the authors use a combination of array tomography and cryoET to describe differences in mitochondrial morphology at the network level and the ultrastructure level between two different cell types in the human placenta. They show that mitochondrial networks are different between the inner cytotrophoblast (CTB) and syncytiotrophoblast (STB), with the network being more tubular in CTB, and more fragmented in STB. Within these mitochondrial networks, they perform cryoET to analyze the cristae architecture and show that CTB mitochondria have lamellar or tubular cristae, whereas the mitochondria in STB have globular cristae. They use proteomics to look at the levels of proteins involved in membrane structure.

Significance: This study is significant because it shows the diversity in mitochondrial shape and structure within the same tissue, suggesting these changes have functional implications for development.

Suggested Areas of Improvement:
1) The primary analysis that appears to be missing from this manuscript is a connection between the proteomics data, which show differences in subunit levels, and the cryoET data, which can provide a direct readout of supercomplex assembly. Without these analyses, the story lacks key evidence that differences in subunits measured by proteomics result in structural assembly differences, as suggested. At the very least, larger complexes, such as ATP synthase and complex I, which have been thoroughly described in other cryo-ET reports and thus facilitate object identification, should be analyzed within their respective tomograms. In my opinion, this would substantially increase the impact of this study beyond a series of somewhat fragmented measurements that hint at, but do not directly test, structure-function relationships across these distinct cell types within the same tissue.

2) The authors also have an opportunity to perform valuable spatial mapping of mitochondrial subtypes in CTB from their array tomography datasets to address the question: Is there a difference in the spatial locations of the two mitochondrial subtypes in CTB (closer or farther away from STB)? What could this mean in terms of differentiation cues and the impact on mitochondria structure and function in these distinct cell types?

3) A previous study (PMID: 25837514) extensively examined the asymmetric partitioning of mitochondria in stem cells and its impact on differentiation. Since previous studies from the same group have shown differences in Complex 1 assemblies between mitochondrial subpopulations, it would be beneficial to compare the proteomics data in the present study with those from prior studies.

4) Since the sample size is relatively low, it would be useful to see graphs plotted for each measurement, separated by placenta (n=2), instead of pooling the data. It would be useful to know how many measurements of each mitochondrial subtype are coming from each placenta to ensure that there is not too much variability, which would require an increase in sample size.

5) I do have some concerns about the reliability of the quantifications, which were mainly performed manually. Mitochondrial membranes can vary in their structure in every direction, making it imperative to make multiple measurements across

different z-planes and regions within the mitochondria to ensure that the differences observed across groups are not due to heterogeneity in mitochondria within the same cell. Several automated approaches exist to reliably segment and analyze membrane ultrastructure from cryo-ET data, enabling more robust statistical analysis. Moreover, the sample size presented in Figure 2 appears to be based on measurements, rather than the number of mitochondria. Further discussion regarding the choice of the various statistical tests used in this study would also help interpret the significance of the quantifications between each experimental group.

6) Both the introduction and discussion sections are somewhat lengthy, focusing on details that are not particularly relevant to the study. There is also considerable speculation in the Discussion section based solely on the proteomic data, which should be avoided or limited.

7) The language in the Discussion section, lines 528-536, does not adequately consider a significant amount of prior art regarding the connection between cristae structure and mitochondrial function. Although cryo-EM has not been performed on these mitochondrial subpopulations, numerous studies have characterized mitochondrial cristae in response to varying cellular energy demands. Moreover, I caution the authors against claiming it is the 'first time' to visualize distinct mitochondria, since there appear to be previous studies in the literature that use room-temperature EM to make similar observations.

Reviewer #2

(Remarks to the Author)
I co-reviewed this manuscript with one of the reviewers who provided the listed reports. This is part of the Nature Communications initiative to facilitate training in peer review and to provide appropriate recognition for Early Career Researchers who co-review manuscripts.

Reviewer #3

(Remarks to the Author)
This is a novel and interesting study comparing mitochondrial structure and levels of key mitochondrial proteins in placental syncytiotrophoblast and cytotrophoblast. The function of these cells is essential for successful pregnancy and offspring health. Cytotrophoblast and syncytiotrophoblast are fundamental to placental function, and the trophoblast is believed to be highly metabolically active, mediating nutrient and waste transport as well as hormone synthesis. There is some evidence that cytotrophoblasts play specific metabolic roles, but this remains poorly understood. This work, providing insight into mitochondrial function in these cells, is therefore very welcome.
The study presents high-resolution imaging at two different scales volume EM of the shapes and spational relationships between mitochondria and TEM tomography of the mitocondrual membranes which is highly infomative. This iamging data provides imprtant context and validation for the proteomics focused on mitochondrial electron transport chain proteins in mitochondria purified from syncytiotrophoblast and cytotrophoblast. The data represent a substantial amount of work, and the alignment between mitochondrial structure and protein levels is impressive.
An acknowledged limitation of the imaging aspect of the study is the sample size. The number of placentas used for imaging should be presented more clearly in the Results in each of the relevant sections. The statistical approach also needs to account for the non-independence of data points; rather than treating each mitochondrion as an independent observation, the placenta (and, for cytotrophoblast, each cell if multiple cells were sampled from the same placenta) should be included as random factors in the statistics.
Enriched fractions of cyto- and syncytio-mitochondria were prepared using previously published methods. The confidence readers can have in the quality of this preparation is central to the confidence they can have in the findings. It would be useful to know how enrichment was assessed and the extent of any cross-contamination.
The proteomics data suggest real functional differences between the two cell types, and it adds weight that these findings could be validated by Western blot.
Specific points:
• The introduction is informative but could be more concise.
• Line 237: Could the number of placentas studied be stated here?
• Line 262: Please clarify whether the two cell types were from different placentas.
• Line 687: The number of placentas is not stated clearly here, unlike for cryo-tomography.
• Line 708: "Mitochondria were identified for segmentation based on the electron density resulting from the double membrane." To make this clear to readers, could the authors point to or add a figure in the text or supplementary material?
• Line 817: Do these tests assume that all data points are independent variables? If so, this is not appropriate where multiple mitochondria come from the same placenta or cell.
• Figure 4: Are full-size Western blots shown in the supplementary material? If not, this would be good practice.
• The conclusion paragraph could be more succinct, with less reptation of results and more on the key messages

Reviewer #4

(Remarks to the Author)
This manuscript describes a descriptive study using array tomography and cryo-electron tomography (cryo-ET) purportedly performed to understand mitochondrial structure in syncytiotrophoblast and their progenitor cytotrophoblast in human

placenta. The emphasis of the manuscript is on using array tomography and cryo-ET in combination to better understand mitochondrial 3D structure in a complex tissue and the changes in mitochondrial structure in relation to the differing functions of cytotrophoblast and syncytiotrophoblast. Yet changes in function in relation to changes in structure are never directly tested experimentally just inferred. Overall the manuscript is very densely written and difficult to wade through with lengthy introduction and discussion. Notably the authors employ mitochondrial isolates from differential centrifugation of whole placental tissue homogenates that they claim to be from cytotrophoblast (CT-mito) and syncytiotrophoblast (syncytio-mitos). Interestingly they do not utilize cell cultures of trophoblast that differentiate in vitro to confirm their findings
The following points need to be addressed:
Methods:
1. The authors employ whole placental tissue homogenates and differential centrifugation to isolate what they claim to be cyto-mito and syncytio-mito. fractions. At term cytotrophoblast comprise only 10% of placental villous tissue and overall trophoblast is only 30% of villous volume, hence a placental homogenate contains mitochondria from several other cell types in addition to trophoblast. Cyto-mito is then perhaps a misnomer? Has this been considered when describing the two distinct populations in the cyto-mito fraction (Lines 299-301)?. Could these be mitochondria from two distinct cell types? Use of trophoblast cell culture where cytotrophoblasts differentiate to syncytiotrophoblast might be illuminating in this regard.
2. Lines 361 -364, it is unclear here that the LC-MS data was generated previously in a different study (described in lines 860-876). This data and the whole of data contained in Figure 3 and Figure 4 again suffers from the fact that Cyto-mito is a mitochondrial population isolated from whole placental tissue, (hence several different cell types) and not just cytotrophoblast cells
Results
1. Figure 3F. Is B actin the correct loading control for western blot of mitochondrial isolates? Should the loading control be a mitochondrial-specific housekeeping protein such as COX IV, VDAC or TOMM20?
2. Figure 3H-K, It is unclear what the authors are trying to represent in these images
3. Additionally the images for the gels and blots are too small in comparison to the heatmaps and proposed models in Figures 3 and 4.
4. The Log2 Fc changes (Fig 3 and 4) do not have a threshold or significance attached to them, which further makes the data shown descriptive in nature.
Discussion
1. The manuscript in its current form is very descriptive and repetitive. Eg the paragraph lines 577 to 593 is a reiteration of what is mentioned in previous paragraphs.
2. The authors seem to be overreaching in some of their conclusions in the discussion. Lines 512-519: the conclusions around mitophagy and aging are all speculation as the authors did not really assess for this in their study.
3. The authors outline that the globular structure of syncytio-mito accounts for larger surface area. However as highlighted in the manuscript, the intermembrane space is important to maintain the proton gradient. Can the authors elaborate on what would be the relevance of the high intermembrane space in syncytio-mitos (lines 549-556)?
4. Were the authors able to confirm the filamentous nature of cyto-mitos in their cryo-ET studies? This is important because the mitochondrial isolation strategy used here is density centrifugation-based which does not necessarily control for the filamentous mitochondria and could potentially result in breakage (and thus alteration in structure).
5. The authors talk about ATP synthase dimers in discussion (lines 561-563) , but this data has not been shown.

Version 1:

Reviewer comments:

Reviewer #1

(Remarks to the Author)
The authors have responded to many of the concerns raised in the review predominantly through textual clarification rather than through new experiments or analyses. While I appreciate the clarification and attempts to address concerns, such as the issues in delineating dimers versus monomers through subtomogram averaging efforts, without new information added to the manuscript, it’s difficult to discern exactly what we have learned beyond what has been described in previous publications. The authors present differences in mitochondria from CTB and STB of the placenta. However, such differences have been noted previously using 2D imaging methods, as mentioned by the authors themselves. The additional in silico analysis of previously published proteomics data (from the same group) reveals differences in mitochondrial complex proteins between the two mitochondrial subtypes. However, similar observations have also already been presented in the previous publication. Without new information, either from mapping changes in macromolecular complexes or from differences in mitochondrial localization across these subtypes, it is hard to see how this paper advances our understanding of placental development beyond what has been shown previously.

Reviewer #2

(Remarks to the Author)
I co-reviewed this manuscript with one of the reviewers who provided the listed reports. This is part of the Nature Communications initiative to facilitate training in peer review and to provide appropriate recognition for Early Career Researchers who co-review manuscripts.

Reviewer #3

(Remarks to the Author)
The manuscript has been revised in line with reviewers suggestions and is an impressive piece of work. I only have a few additional suggestions.

Public accessibility of image data is important for future validation and reuse. In addition to inclusion within the source data file the raw image data figures should also be uploaded to a publicly available online repository such as bioimage archive (https://www.ebi.ac.uk/bioimage-archive/). These repositories represent a more searchable and accessible than accompanying data to a paper facilitating access and reuse. It is not clear why the image reconstructions could not also be uploaded here.

Line 150: “ideal model” is a bold claim and could be rephrased.

In figure 1 I and L there are a couple of points with near zero values, out of keeping with the distribution of the rest of the data. Are the author’s certain these represent true mitochondria?

The authors could also consider referring to “mitos” as “mitochondria”

Reviewer #4

(Remarks to the Author)
The authors have addressed the majority of comments appropriately and the manuscript is now more easily readable. This reviewer however is still concerned by use of the term cyto-mito to describe the preparation used. Despite arguments that cytotrophoblast may comprise more than 10% of placental tissue at term the authors do not acknowledge that their cyto-mito preparation in all probability does contain mitochondria from other placental cell types including, endothelial cells, stroma, smooth muscle and macrophages, as they homogenize whole villous tissue. They present a robust defense of their preparation and cite other manuscripts in support. In examining these however the Kolahi et al 2017 paper does not use mitochondrial preparations, and the Martinez et al 1997 paper uses the technique only to prepare highly enriched syncytiotrophoblast mitochondria for study and does not study the remaining (cytotrophoblast + other) mitochondria from the preparation. The authors used the Martinez technique and alone invented the terminology cyto-mito but have never acknowledged the preparation probably contains more than cyto-mito. Statements about the number of citations do not negate this argument. Further arguments related to purity of preparations are an obfuscation here as purity of a mitochondrial preparation does not relate to derivation from different cell types. Can the authors present data showing the cellular origin of their preparation?

Version 2:

Reviewer comments:

Reviewer #4

(Remarks to the Author)
The imaging data supplied (array tomography and cryo-ET) clearly shows the distinction between cytotrophoblast and syncytiotrophoblast mitochondria and the supplemental data in Fig 14 shows distinction in size of mitochondria from hofbauer and endothelial cells in the preparation. The preparations used for the proteomic analysis and the immunoblot and western blot analyses likely however contain contributions from other villous tissue cell types. While the authors now acknowledge this limitation this may be more than "minor" as 60% of villous tissue volume is not trophoblast.

Open Access This Peer Review File is licensed under a Creative Commons Attribution 4.0 International License, which permits use, sharing, adaptation, distribution and reproduction in any medium or format, as long as you give appropriate credit to the original author(s) and the source, provide a link to the Creative Commons license, and indicate if changes were made.
In cases where reviewers are anonymous, credit should be given to 'Anonymous Referee' and the source.
The images or other third party material in this Peer Review File are included in the article's Creative Commons license, unless indicated otherwise in a credit line to the material. If material is not included in the article's Creative Commons license and your intended use is not permitted by statutory regulation or exceeds the permitted use, you will need to obtain permission directly from the copyright holder.
To view a copy of this license, visit https://creativecommons.org/licenses/by/4.0/

Reviewer #1 (Remarks to the Author):

Summary: In this study, the authors use a combination of array tomography and cryoET to describe differences in mitochondrial morphology at the network level and the ultrastructure level between two different cell types in the human placenta. They show that mitochondrial networks are different between the inner cytotrophoblast (CTB) and syncytiotrophoblast (STB), with the network being more tubular in CTB, and more fragmented in STB. Within these mitochondrial networks, they perform cryoET to analyze the cristae architecture and show that CTB mitochondria have lamellar or tubular cristae, whereas the mitochondria in STB have globular cristae. They use proteomics to look at the levels of proteins involved in membrane structure.

Significance: This study is significant because it shows the diversity in mitochondrial shape and structure within the same tissue, suggesting these changes have functional implications for development.

Response:

We thank the reviewer for acknowledging the significance of the work performed, and for providing detailed feedback and suggestions for improving this manuscript. We have endeavoured to implement these suggestions in our resubmission and have provided responses to the comments below.

Reviewer #1 (Remarks to the Author):

Suggested Areas of Improvement:

1) The primary analysis that appears to be missing from this manuscript is a connection between the proteomics data, which show differences in subunit levels, and the cryoET data, which can provide a direct readout of supercomplex assembly. Without these analyses, the story lacks key evidence that differences in subunits measured by proteomics result in structural assembly differences, as suggested. At the very least, larger complexes, such as ATP synthase and complex I, which have been thoroughly described in other cryo-ET reports and thus facilitate object identification, should be analyzed within their respective tomograms. In my opinion, this would substantially increase the impact of this study beyond a series of somewhat fragmented measurements that hint at, but do not directly test, structure-function relationships across these distinct cell types within the same tissue.

Response:

We acknowledge that this additional experimental data would provide great insight into the structure of ATP synthase and the electron transport chain, and demonstrate their ability to induce membrane curvature. Indeed, we ourselves and other mitochondrial biologists within the field of cryo-ET are intrigued by this very question, particularly with respect to human mitochondria.

We have been able to build an initial model of monomeric ATP synthase from particle picks ($n \leq 100$), however current applications of subtomogram averaging warrant several thousand to hundreds of thousands of particle picks to build high-resolution protein structures. For our data, we found that the mitochondrial matrix was too dense to gain sufficient visibility of ATP

synthase particles, particularly in dimeric form. Even for the particles that were picked, the background noise of the matrix significantly impacted the model generation of ATP synthase, forcing excessive binning and increased thresholding, a challenge particularly evident in the Syncytio-Mito populations. Without the ability to produce subtomogram averages of ATP synthase dimers or supercomplexes, we decided to withhold this data from this particular manuscript to ensure that our conclusions remain robust and could not be misinterpreted. Moreover, this issue led to the publication of a review (https://doi.org/10.1016/j.placenta.2026.01.009) extensively discussing the necessity of a focused-ion-beam SEM (FIB-SEM) to produce lamellae for performing subtomogram averaging. In the absence of FIB-SEM methodology for milling heterogeneous human tissues, this study chose to observe isolated mitochondria.

Reviewer #1 (Remarks to the Author):

2) The authors also have an opportunity to perform valuable spatial mapping of mitochondrial subtypes in CTB from their array tomography datasets to address the question: Is there a difference in the spatial locations of the two mitochondrial subtypes in CTB (closer or farther away from STB)? What could this mean in terms of differentiation cues and the impact on mitochondria structure and function in these distinct cell types?

Response:

We, like the reviewer, are very intrigued by this question, whether there is any correlation between mitochondrial size and proximity to the CTB versus proximity to the intervillous space/shedding side of the syncytium. We are particularly interested in this, given our observations of what may be a transitioning population of mitochondria with mixed cristae morphology. Indeed, even with our own array tomography imaging, it is clear that there is a high degree of morphological diversity across CTB and STB mitochondria, as shown in Figure 1. However, the challenge at present is the CTB proximity is far more complex that initially observed as it is a construct of the field (ourselves included) to think in a 2D plane rather than the 3D space, i.e. sections of the STB may appear distal from the CTB at a single point, however through 3D reconstruction, the continual nature of the cells appears closer or aligned with several CTB. We are unfortunately unable to speculate at present time on what these cues may infer.

Reviewer #1 (Remarks to the Author):

3) A previous study (PMID: 25837514) extensively examined the asymmetric partitioning of mitochondria in stem cells and its impact on differentiation. Since previous studies from the same group have shown differences in Complex 1 assemblies between mitochondrial subpopulations, it would be beneficial to compare the proteomics data in the present study with those from prior studies.

Response:

We thank the reviewer for highlighting this insightful study, and for referring us to the group's previous work. We find this data to be valuable and relevant to our work, as it demonstrates the role of mitochondria in determining cell differentiation direction and fate. We were intrigued by Dohla et al.'s 2022 paper from the same group finding that the majority of ETC subunits

were more abundant in the stem-like mitochondria compared to differentiated cells. The specific names of the subunits were not provided in Dohla et al.'s publication and thus unable to compare the proteomics data, and we did not observe such a distinct trend, but instead found equal variance of ETC subunits among the progenitor CTBs and the differentiated STB. We have cited and discussed Dohla et al.'s paper in our discussion (now line 525). In line with this study and it's discussion; we have also cited this recently published review (https://doi.org/10.1242/jcs.263847) (now line 435).

Reviewer #1 (Remarks to the Author):

4) Since the sample size is relatively low, it would be useful to see graphs plotted for each measurement, separated by placenta (n=2), instead of pooling the data. It would be useful to know how many measurements of each mitochondrial subtype are coming from each placenta to ensure that there is not too much variability, which would require an increase in sample size.

Response:

We have now included all array tomography graphs separated by patient, in a supplementary file (Supplementary Fig. 10). Crucially, we maintained significant differences between CTB and STB mitochondria populations, across volume, surface area, and L:W, regardless of being separated by patient and subsequent reduction in the group-specific sample size. Significance was observed between placenta samples in some assessments between the same cell populations across different placentas (CTB vs CTB; STB vs STB), although, this was not the focus of this study. Critically, significance was maintained between CTB and STB irrespective of placental variance and reduced samples size, a finding that strengthens our conclusions.

We note that the Kruskal-Wallis ANOVA with Dunn's multiple comparisons test was used to compare between groups for the purpose of visualising differences between placentae in this supplementary figure, however to account for the non-independence of data points as suggested by Reviewer #3, we have now incorporated a Linear Mixed Model Analysis into the main methodology of this study, shown now in Figure 1 and Supplementary Fig. 4-9. This analysis considers the cell population as a fixed effect, and the placental sample as a random intercept to account for clustering. This analysis shows that irrespective of placental sample (accounting for between 0-19% of variance), cell population remained a significant predictor of mitochondrial variation between CTBs and the STB ($p < .001$).

Reviewer #1 (Remarks to the Author):

5) I do have some concerns about the reliability of the quantifications, which were mainly performed manually. Mitochondrial membranes can vary in their structure in every direction, making it imperative to make multiple measurements across different z-planes and regions within the mitochondria to ensure that the differences observed across groups are not due to heterogeneity in mitochondria within the same cell. Several automated approaches exist to reliably segment and analyze membrane ultrastructure from cryo-ET data, enabling more robust statistical analysis. Moreover, the sample size presented in Figure 2 appears to be based on measurements, rather than the number of mitochondria. Further discussion regarding the

choice of the various statistical tests used in this study would also help interpret the significance of the quantifications between each experimental group.

Response:

We have amended the intermembrane distance and cristae angle graphs (Figure 2) to present mean values per mitochondrion or per cristae respectively, and these values have now been used for the statistical analysis.

To address the challenges associated with measuring across different Z-planes and regions within mitochondria and the measurement of angles, we complimented these measurements with a standardised metric of 3D membrane curvature, which assesses angularity across all Z-planes and regions of cristae and is not subject to individual manual measurements (demonstrated in https://doi.org/10.1002/cne.25254).

We acknowledge the reviewer's concern regarding manual quantifications of cryo-ET data, however in this study, we employed the reliability and reproducibility of interpolation-assisted manual segmentation, whereby the majority of segmentations are generated by predicting the location of the membrane between interspaced manual assessments (https://doi.org/10.1111/jmi.12676). We have ensured that our approach has remained accurate by using a combination of interpolation, threshold-based membrane detection and segmentation, and inspection in all three axes. We also ensured that a consistent approach was used for each distance measurement, and opted for a four-quadrant multi-measurement system where several measurements of different regions were averaged to prevent bias and limit the effects of single-mitochondrion heterogeneity. Additionally, all length and width measurements were recorded as maximal values and inspected across different axes and viewing angles. We have summarised this measurement approach in the methods and supplementary figure (Supplementary Fig. 3).

Indeed, automated segmentation tools do exist, however at present, these introduce other significant challenges within our dataset. Notably, Membrain v2 is one such tool we initially used, which also has a classification of connected components feature, however it was unable to detect and segment deeper and less visible membranes due to the noise and density of the mitochondrial matrix, leading to fragmented and partially segmented cristae, particularly the case for dense Syncytio-Mitos. The resulting mesh holes would require extensive manual clean up and interpolation, thereby introducing less control, more variation, and unintentional bias into the dataset. These artefacts were avoided by performing interpolation-assisted manual segmentation.

Regarding the statistical tests used, in line with Reviewer #3, we have now incorporated linear mixed model analysis of all array tomography data, with cell population as a fixed factor, and placental sample as a random intercept to account for clustering. We believe that this method best accounts for the non-independence of data points, and have discussed this further in the methodology section (now line 761).

Reviewer #1 (Remarks to the Author):

6) Both the introduction and discussion sections are somewhat lengthy, focusing on details that are not particularly relevant to the study. There is also considerable speculation in the Discussion section based solely on the proteomic data, which should be avoided or limited.

Response:

We have now significantly amended the introduction, and reduced it from 1773 to 940 words. Subsequent refinement of the discussion has also condensed and linearised our findings, reducing the length from 2849 to 2510 words. We acknowledge the reviewer's suggestion to avoid speculation in the discussion, and have therefore removed sections that were overstated and delineated our findings from hypotheses inferred from the data.

Reviewer #1 (Remarks to the Author):

7) The language in the Discussion section, lines 528-536, does not adequately consider a significant amount of prior art regarding the connection between cristae structure and mitochondrial function. Although cryo-EM has not been performed on these mitochondrial subpopulations, numerous studies have characterized mitochondrial cristae in response to varying cellular energy demands. Moreover, I caution the authors against claiming it is the 'first time' to visualize distinct mitochondria, since there appear to be previous studies in the literature that use room-temperature EM to make similar observations.

Response:

We acknowledge and apologise that our sentiment was not conveyed appropriately, and we have therefore removed areas of text that are overstated or that may be seen to disregard previous studies through the language chosen. It was not our intention to disregard previous studies that have contributed greatly to our understanding of cristae structure and bioenergetic function. Our statement was specifically regarding the analysis and comparison of 3D cristae curvedness between multiple cristae pockets within a singular mitochondrion, and between other mitochondria from other cells, which to our knowledge, this study is the first to complete.

Reviewer #2 (Remarks to the Author):

I co-reviewed this manuscript with one of the reviewers who provided the listed reports. This is part of the Nature Communications initiative to facilitate training in peer review and to provide appropriate recognition for Early Career Researchers who co-review manuscripts.

Response:

As an ECR led manuscript (first and senior author), we appreciate the reviewer's commitment to ECR development and for providing feedback and suggestions alongside the additional reviewer.

Reviewer #3 (Remarks to the Author):

This is a novel and interesting study comparing mitochondrial structure and levels of key mitochondrial proteins in placental syncytiotrophoblast and cytotrophoblast. The function of these cells is essential for successful pregnancy and offspring health. Cytotrophoblast and

syncytiotrophoblast are fundamental to placental function, and the trophoblast is believed to be highly metabolically active, mediating nutrient and waste transport as well as hormone synthesis. There is some evidence that cytotrophoblasts play specific metabolic roles, but this remains poorly understood. This work, providing insight into mitochondrial function in these cells, is therefore very welcome.

The study presents high-resolution imaging at two different scales volume EM of the shapes and spational relationships between mitochondria and TEM tomography of the mitocondrual membranes which is highly infomative. This iamging data provides imprtant context and validation for the proteomics focused on mitochondrial electron transport chain proteins in mitochondria purified from syncytiotrophoblast and cytotrophoblast. The data represent a substantial amount of work, and the alignment between mitochondrial structure and protein levels is impressive.

Response:

We thank the reviewer for their kind comments regarding the alignment of imaging modalities and protein assessments, and for sharing our interest in the fundamental unanswered questions related to trophoblast bioenergetics.

Reviewer #3 (Remarks to the Author):

An acknowledged limitation of the imaging aspect of the study is the sample size. The number of placentas used for imaging should be presented more clearly in the Results in each of the relevant sections. The statistical approach also needs to account for the non-independence of data points; rather than treating each mitochondrion as an independent observation, the placenta (and, for cytotrophoblast, each cell if multiple cells were sampled from the same placenta) should be included as random factors in the statistics.

Response:

We have now added the number of placentas used for each methodology, within the results section (now line 173; line 229; line 297; line 315; line 371). We have also amended the intermembrane space and cristae angle graphs in Figure 2 so that each data point represents a mean value per mitochondrion or per cristae respectively, rather than presenting all measurements as individual data points. These mean values are now also used in the statistical analysis within Figure 2.

To address concerns around non-independence of data points, we have now presented our array tomography data as a linear mixed model, with mitochondrial measurements (surface area, volume, SA:V, elongation, sphericity, and MCI) as dependent variables, cell population (CTB or STB) as a fixed effect, and placenta as a random intercept to account for clustering. Since mitochondria were derived from one CTB and a region of STB for each placenta, we do not need to account for cell as a fixed factor.

Critically, in line with our interpretation, presentation, and discussion of results, cell population remained a significant predictor across all volumetric parameters, even after accounting for placental clustering (all measurements $p < .001$). This provides great confidence that our interpretation and discussion of the results appropriately considers the nesting of variables and

the comparison of volumetrics between CTB and STB mitochondria. Volume and surface area demonstrated the largest effect sizes ($F > 150$), and placental-level clustering accounted for between 0-19% variance, depending on the parameter. This is further elaborated below:

**Measurement**	**Cell population predicted significance**	**Variance attributable to placental difference**
Surface area	$P < .001$	19%
Volume	$P < .001$	17%
SA:V	$P < .001$	1%
XY elongation	$P < .001$	3%
Sphericity	$P < .001$	6%
Mitochondrial complexity index (MCI)	$P < .001$	7%

These statistical findings have now been included in Figure 1, replacing the previous tests. We have also included further clarity on this linear mixed effects model analysis, performed in SPSS, in the methods section (now line 762), and the statistical report is included in the supplementary files (Supplementary Fig. 4-9).

Reviewer #3 (Remarks to the Author):

Enriched fractions of cyto- and syncytio-mitochondria were prepared using previously published methods. The confidence readers can have in the quality of this preparation is central to the confidence they can have in the findings. It would be useful to know how enrichment was assessed and the extent of any cross-contamination.

Response:

The mitochondrial isolation method is a commonly utilised protocol first established by Martinez et al., that was adapted to maintain functional bioenergetic characteristics (i.e. the mitochondria continue to respire and produce ATP), and has subsequently been used in this study. This method is well-established within the placental field, with the original publication by Martinez et al., being cited 90 times since initial publication, and 9 times since 2024. Enrichment was and is continuously assessed within the field by quantitative imaging, mitochondrial copy number counts through PCR ratios, immunofluorescence staining, proteomic analysis, western blotting, and functional assessments including metabolic assays and respirometry in both healthy and pathological placentas (gestational diabetes mellitus, fetal growth restriction, across trimesters from multiple laboratory groups)(https://doi.org/10.1038/srep42941; https://doi.org/10.1210/endo.138.5.5133; https://doi.org/10.1016/j.placenta.2019.05.004; https://doi.org/10.1113/JP280593). The isolation protocol has also been validated to produce enriched fractions of Cyto- and Syncytio-Mitos with up to 95% purity (https://doi.org/10.1016/j.placenta.2019.05.004), and cited 38 times since publication. However, we do acknowledge that there is always room for improvement, and have several methods currently under development to advance the isolation quality, quantity, and reduce contaminants that are inherently present. Most notably, all our existing understanding of the differences in bioenergetic functionality between Cyto- and Syncytio-Mitos is derived from analyses of mitochondrial isolates produced through this same

protocol. For our cryo-ET examinations, we wished to contribute to the existing body of literature by also using this isolation protocol, to support prior functional assessments with our structural analyses. We have now added further clarification of these points in our limitations section (now line 574).

Across other fields, mitochondrial isolation continues to be a standard practice for cryo-ET, across cell types and species, including humans (demonstrated in https://doi.org/10.1016/j.str.2017.07.011) and a wide range of unicellular organisms (https://doi.org/10.1042/BCJ20230450).

Reviewer #3 (Remarks to the Author):

The proteomics data suggest real functional differences between the two cell types, and it adds weight that these findings could be validated by Western blot.

Response:

We thank the reviewer for their comment and agree on the importance of western blot validation of proteomics results in additional samples to ensure a robust, reproducible interpretation of data. We have now also provided full-size western blots in the supplementary files (Supplementary Fig. 11-13).

Reviewer #3 (Remarks to the Author):

Specific points:

• The introduction is informative but could be more concise.

Response:

We agree with the reviewer, and this has been amended by reducing the introduction word count from 1773 to 940 words.

Reviewer #3 (Remarks to the Author):

• Line 237: Could the number of placentas studied be stated here?

Response:

We have now stated the number of placentas studied by array tomography (now line 173).

Reviewer #3 (Remarks to the Author):

• Line 262: Please clarify whether the two cell types were from different placentas.

Response:

We have now clarified that the subset was randomly selected from the full array tomography sample size (CTB and STB each from 2 placentas), now line 213 and line 217.

Reviewer #3 (Remarks to the Author):

• Line 687: The number of placentas is not stated clearly here, unlike for cryo-tomography.

Response:

Samples were derived from two placentae of healthy full-term pregnancies. This has been stated in line 173 for array tomography and line 229 for cryo-ET.

Reviewer #3 (Remarks to the Author):

• Line 708: “Mitochondria were identified for segmentation based on the electron density resulting from the double membrane.” To make this clear to readers, could the authors point to or add a figure in the text or supplementary material?

Response:

We have now included an example of CTB and STB mitochondria from our array tomography data in the supplementary file (Supplementary Fig. 1).

Reviewer #3 (Remarks to the Author):

• Line 817: Do these tests assume that all data points are independent variables? If so, this is not appropriate where multiple mitochondria come from the same placenta or cell.

Response:

In line with the previous comment about the non-independence of data points, we have now incorporated the linear mixed model analysis of array tomography data, with cell population (CTB or STB) as a fixed factor and placental sample as a random intercept to account for clustering. Since mitochondria were derived from only one CTB and a region of STB for each placenta, we have not accounted for cell as a fixed factor. The statistical significance shown on Figure 1G-L have been adjusted to represent our findings from the linear mixed model analysis. Critically, our finding that cell population governs mitochondrial structural differences, is retained in line with our interpretation of the results and discussion.

Reviewer #3 (Remarks to the Author):

• Figure 4: Are full-size Western blots shown in the supplementary material? If not, this would be good practice.

Response:

We agree with the reviewer that full-size blots should be provided to improve the transparency of results, and have now included all full-size blots in the supplementary file (Supplementary Fig. 11-13).

Reviewer #3 (Remarks to the Author):

• The conclusion paragraph could be more succinct, with less reptation of results and more on the key messages

Response:

We have now made the conclusion more concise, reducing the word count from 286 to 185 words.

Reviewer #4 (Remarks to the Author):

This manuscript describes a descriptive study using array tomography and cryo-electron tomography (cryo-ET) purportedly performed to understand mitochondrial structure in syncytiotrophoblast and their progenitor cytotrophoblast in human placenta. The emphasis of the manuscript is on using array tomography and cryo-ET in combination to better understand mitochondrial 3D structure in a complex tissue and the changes in mitochondrial structure in relation to the differing functions of cytotrophoblast and syncytiotrophoblast. Yet changes in function in relation to changes in structure are never directly tested experimentally just inferred. Overall the manuscript is very densely written and difficult to wade through with lengthy introduction and discussion. Notably the authors employ mitochondrial isolates from differential centrifugation of whole placental tissue homogenates that they claim to be from cytotrophoblast (CT-mito) and syncytiotrophoblast (syncytio-mitos). Interestingly they do not utilize cell cultures of trophoblast that differentiate in vitro to confirm their findings

Response:

We thank the reviewer for their comments and have endeavoured to refine the manuscript to improve readability. While function was not directly assessed within this manuscript, this is due to the extensive publications within the field, including by us, that have already performed and published these findings, and it felt disingenuous to repeat the work in another publication (https://doi.org/10.1038/srep42941; https://doi.org/10.1210/endo.138.5.5133; https://doi.org/10.1016/j.placenta.2019.05.004; https://doi.org/10.1113/JP280593). Thus, we have referenced these reproducible findings extensively throughout the manuscript.

In regard to the utilisation of cell culture, in vitro models were not used in this study as we believe they are not best representative of native trophoblasts derived from placental villi, nor the mitochondria therein residing in the native cytotrophoblast and syncytiotrophoblast environment. While great strides have been made within in vitro culture and organoid models, we and others in the field are still analysing whether the mitochondrial phenotypes and network structures are maintained in such models, or representative of their in vivo phenotype.

Methodologically, there are also limitations to such approaches, including the challenges associated with growing organoids on EM grids for cryo-ET, given the propensity for cells to attach to grid bars, which are not able to be imaged by cryoTEM. In lieu of this, our study decided to incorporate a multimodal imaging approach, by utilising in situ network imaging (array tomography), and assess isolated mitochondria (cryo-ET) as a foundation for future in vitro works to build upon.

Moreover, the extensive methodological development required for fluorescence-guided FIB-SEM milling of lamellae within cultured organoids is beyond the scope of this manuscript. Given the importance and demand for such a method, we believe such an advancement would constitute a separate, high-impact publication in its own right.

Reviewer #4 (Remarks to the Author):

The following points need to be addressed:

Methods:

1. The authors employ whole placental tissue homogenates and differential centrifugation to isolate what they claim to be cyto-mito and syncytio-mito. fractions. At term cytotrophoblast comprise only 10% of placental villous tissue and overall trophoblast is only 30% of villous volume, hence a placental homogenate contains mitochondria from several other cell types in addition to trophoblast. Cyto-mito is then perhaps a misnomer? Has this been considered when describing the two distinct populations in the cyto-mito fraction (Lines 299-301)?. Could these be mitochondria from two distinct cell types? Use of trophoblast cell culture where cytotrophoblasts differentiate to syncytiotrophoblast might be illuminating in this regard.

Response:

We wish to highlight that the idea that cytotrophoblasts comprise only 10% of villous volume has been derived from 2D electron microscopy of placental sections, and we suggest that this value is likely to be greater based on our 3D array tomography imaging, particularly for terminal villi that consist of less stromal tissue. In 2D, cytotrophoblasts appear sporadically and are not frequent, but in 3D, many cytotrophoblasts can be seen in the Z-depth. Indeed, there are less cytotrophoblasts than would be seen in the 1st or 2nd trimester, where the entire basement membrane is lined with the trophoblast bilayer. We believe that the notion of 10% may be an underestimation that has been entrenched in dogma within the placental field, resulting from extensive 2D imaging. This is a factor we discuss in depth in a recently published, invited review, in addition to future considerations for the field (https://doi.org/10.1016/j.placenta.2026.01.009).

The mitochondrial isolation protocol we have used is well established in the placental research field as a method to assess and quantify CTB and STB mitochondria. Several studies have used this protocol to examine functionality between CTB and STB mitochondrial populations, identifying differences in respiratory capacity, protein abundance, steroidogenic capacity, and morphology between CTB and STB mitochondria (Kolahi et al., 2017; Fisher et al., 2019; Martinez et al., 1997). We do acknowledge however, that this protocol does not produce 100% pure isolations, but results in enriched fractions, with up to 95% purity (Fisher et al., 2019). While ultracentrifugation may be used to produce pure fractions, the resulting complete loss of mitochondrial functionality, respiratory capacity, and impacts to cristae structure, are features of this methodology we wished to avoid.

As such, we have chosen to incorporate a multimodal imaging approach to address this question, by complementing our cryo-ET data of isolated mitochondria with array tomography data of mitochondria within intact placental tissue. We acknowledge that the development of a

specific marker between the two populations may enable more precise localisation through adjacent advanced imaging methods.

We have chosen not to perform cryo-ET on cells for the reasons outlined in our prior response. Notably cell models do not currently recapitulate the physiology and complex ultrastructure of the villous trophoblast bilayer from which our mitochondrial isolates are derived.

Reviewer #4 (Remarks to the Author):

2. Lines 361 -364, it is unclear here that the LC-MS data was generated previously in a different study (described in lines 860-876). This data and the whole of data contained in Figure 3 and Figure 4 again suffers from the fact that Cyto-mito is a mitochondrial population isolated from whole placental tissue, (hence several different cell types) and not just cytotrophoblast cells

Response:

We apologise for the confusion regarding this statement and have amended this in text, "…in silico analysis of the previously published proteomic dataset…". We can clarify that the LC-MS dataset was generated in a previous study, and proteins that were identified to be the most highly altered between populations using Fisher's exact test were previously published. For this manuscript, we performed an in silico analysis of the previously generated LC-MS dataset exclusively focusing on proteins within our pathways of interest that were not published in the original manuscript. Only five proteins were excluded from our proteins of interest as a result of previous publication, ATP5β, ATP5α, SDHA, NDUFA12, and MIC19, the implications of which are included and referenced in the discussion and many of these findings formed the basis for this study itself.

We continue to believe that the use of mitochondrial isolates instead of a cytotrophoblast cell model (such as Okae/human trophoblast stem cells) has specific benefits, given these cells have been shown to have a different transcriptomic profile when compared to primary cytotrophoblast cells from tissue (https://doi.org/10.1016/j.placenta.2024.11.007). Moreover, the chemically induced differentiation protocols present in some models to generate STB, likely do not represent the physiological processes involved in trophoblast differentiation, particularly in the case of ROCK-inhibited differentiation and forskolin-driven cAMP stimulation. Similarly, primary trophoblast isolation and culture in vitro likely does not represent the in situ environment, as a result of isotonic stress, shear stress, and different substrate profiles. The impact of culture conditions on ATP consumption relative to the in vivo environment has been established to differ, based on the type of trophoblast cells, the trimester from which they were obtained, and culture length. As discussed in the follwoing referenced paper, trophoblast cells show significant metabolic flexibility and can switch between glycolysis and oxidative phosphorylation depending on environmental conditions (in vitro, in vivo, explants, isolated cells) (https://doi.org/10.1093/biolre/ioaf273). Whereas the mitochondrial isolates used in our manuscript are isolated and cryopreserved within 30 minutes of delivery, capturing we believe, the best representation of the in situ environment possible with current methodologies.

Reviewer #4 (Remarks to the Author):

Results

1. Figure 3F. Is B actin the correct loading control for western blot of mitochondrial isolates? Should the loading control be a mitochondrial-specific housekeeping protein such as COX IV, VDAC or TOMM20?

Response:

We acknowledge the field's use of mitochondrial-specific housekeeper proteins such as VDAC and TOMM20, but we made the conscious decision not to use these proteins as they are inherently dependant on mitochondrial membrane surface area, which we have noted in this study to be changed between populations, as shown by our array tomography imaging (Fig. 1). Similarly, COX IV was also not used as many subunits have been noted in our study to change across mitochondrial populations from the CTB and STB (Fig. 4). As the suggested proteins are dependent on variables, we believe they do not in this instance make good housekeepers. GAPDH is one such other protein commonly used as a mitochondrial housekeeper, however in our own initial usage we observed substantial variation between groups and chose instead to use Beta Actin, which remains consistent and reproducible across numerous western blots in several studies, including those in preprint and previously published (such as https://doi.org/10.3389/fphys.2019.01536).

Reviewer #4 (Remarks to the Author):

2. Figure 3H-K, It is unclear what the authors are trying to represent in these images

Response:

The images in Fig. 3H-K are intended to represent the structural differences between cristae junctions of Cyto- and Syncytio-Mitos, as observed by cryo-ET. We placed H-K below our diagram of the cristae organisation proteins (MICOS, SAM, MTX) to illustrate that while Cyto-Mitos have a highly organised cristae junction alongside high abundance of MICOS, the loss of MICOS in Syncytio-Mitos also corresponds to the collapse of lamellar cristae and the flattening of the inner mitochondrial membrane. To clarify this, we have amended the text in the results section (now line 318).

Reviewer #4 (Remarks to the Author):

3. Additionally the images for the gels and blots are too small in comparison to the heatmaps and proposed models in Figures 3 and 4.

Response:

We have now provided full-size western blots in the supplementary files (Supplementary Fig. 11-13). We have also slightly increased the size of the blots in Figure 3 and 4, and reduced the size of Figure 4J, however we have not reduced the size of the heatmaps and proposed models as it may impair the visibility and readability of the small text labels of individual subunit locations.

Reviewer #4 (Remarks to the Author):

4. The Log2 Fc changes (Fig 3 and 4) do not have a threshold or significance attached to them, which further makes the data shown descriptive in nature.

Response:

In this manuscript, we conducted an in silico analysis of an existing proteomics dataset (expanded upon above). No threshold was implemented as we were not intending to observe the proteins with the greatest statistical change (as the initial publication generated from this dataset and others provide), but rather compare specific proteins of interest.

The use of thresholds such as 2 or 1.5 is important for determining pathways, mechanisms, or proteins of interest from initial screens, and has been instrumental in advancing our knowledge and developing a standardised method for discovery. However, thresholding reveals only a very restricted aspect of the whole dataset, and may leave smaller intricate and physiological changes with biological relevance undiscovered, a limitation induced by large datasets across many "omics" platforms (https://doi.org/10.20900/mo.20170002; https://doi.org/10.1038/s10038-020-0763-5).

Examination of the dataset with a focus on directionality and analysis between groups should not be overlooked. For example, a Log2FC of 0.5 may fall under a given threshold, although represents a 41.4% increase, which within controlled and well-defined pathways, likely has a biological relevance. This is particularly relevant, given our confirmation of the LC-MS data by western blotting of specific proteins of interest. For example, in Figure 4, a 0.19 Log2FC change in ATP5MK showed statistically and biologically greater differences in protein levels in the cytotrophoblast when assessed by western blotting.

Reviewer #4 (Remarks to the Author):

Discussion
1. The manuscript in its current form is very descriptive and repetitive. Eg the paragraph lines 577 to 593 is a reiteration of what is mentioned in previous paragraphs.

Response:

We have amended the document and have reduced the introduction from 1773 to 940 words and condensed and linearised our findings in the discussion, from 2849 to 2510 words.

Reviewer #4 (Remarks to the Author):

2. The authors seem to be overreaching in some of their conclusions in the discussion. Lines 512-519: the conclusions around mitophagy and aging are all speculation as the authors did not really assess for this in their study.

Response:

Our comments regarding the potential role of mitochondrial network fragmentation in the STB is indeed a theory that we propose in response to our observations of network structure and

underpinning protein dynamics and prior literature supporting similar roles in other tissues. We acknowledge that speculations should remain as such and not be overstated, and have now amended line 443, to include "…however further analysis is required to assess links between spatial distribution and network structure…". We have also amended the discussion as a whole to appropriately discuss the findings without overstating.

Reviewer #4 (Remarks to the Author):

3. The authors outline that the globular structure of syncytio-mito accounts for larger surface area. However as highlighted in the manuscript, the intermembrane space is important to maintain the proton gradient. Can the authors elaborate on what would be the relevance of the high intermembrane space in syncytio-mitos (lines 549-556)?

Response:

As Syncytio-Mitos are not functionally required to produce as much ATP as Cyto-Mitos (as shown in other studies by their reduced respiratory capacity), they likely do not depend upon tightly constricted intermembrane spaces that ordinarily yield higher proton densities and greater electrochemical gradients, whereas these structural features are present in highly bioenergetic Cyto-Mitos.

We propose that Syncytio-Mitos have a high intermembrane space for two reasons:

i. The withdrawal of MIB complexes collapses cristae junctions, forming a high intermembrane space to accommodate reduced bioenergetic demands in the differentiated STB. The increased intermembrane space thereby reduces proton density (protons per cubic volume), resulting in reduced flux of protons through ATP synthase.

ii. Functional specialisation of Syncytio-Mitos for steroidogenic capacity, whereby a greater intermembrane space facilitates efficient diffusion and shuttling of cholesterol to the inner membrane, where P450scc synthesises pregnenolone (https://doi.org/10.1210/endo.138.5.5133; https://doi.org/10.1210/en.2005-1204). Moreover, cristae junctions are observed to be diffusion barriers, tightening the inner mitochondrial membrane and forming closed cristae lumen for the storage of protons. The presence of cristae junctions would make large regions of the inner membrane inaccessible to cholesterol, and conversely, we propose that absence of such junctions in Syncytio-Mitos provides greater surface area for the placement of P450scc, increasing steroidogenic capacity in the STB.

While this is the proposed relevance of intermembrane space in Syncytio-Mitos, we acknowledge in text (now line 464) that further study is necessary to confirm this.

Reviewer #4 (Remarks to the Author):

4. Were the authors able to confirm the filamentous nature of cyto-mitos in their cryo-ET studies? This is important because the mitochondrial isolation strategy used here is density centrifugation-based which does not necessarily control for the filamentous mitochondria and could potentially result in breakage (and thus alteration in structure).

Response:

We are yet to observe filamentous-like mitochondria in our cryo-ET studies, which we attribute to the disruption of mitochondrial networks by centrifugation. At present, cryo-ET is limited by its field of view, whereby increasing the magnification and resolution reduces the area that can be imaged. To image such large and filamentous and networked mitochondria, would require a larger imaging area, as provided by array tomography (Figure 1).

Moreover, we refer the reviewer to our published review (https://doi.org/10.1016/j.placenta.2026.01.009) that discusses these limitations and potential solutions in more detail. This is also incorporated into the limitations section (now line 577). In short, in the absence of in-situ lamella milling within tissue (FIB-SEM), these observations cannot be obtained. In the absence of this methodology, we have chosen to incorporate a multimodal imaging approach, uniting tissue imaging by array tomography alongside higher resolution imaging of isolated mitochondria by cryo-ET. Due to the thickness of cells or tissue obstructing the transmission of electrons during cryo-ET, we have chosen to use thinner enriched and isolated fractions of Cyto- and Syncytio-Mitos instead. We believe that this is an important step towards understanding mitochondrial structure and its underlying mechanisms, and the establishment of methodologies within the placental field that do not currently exist in the literature.

Reviewer #4 (Remarks to the Author):

5. The authors talk about ATP synthase dimers in discussion (lines 561-563) , but this data has not been shown.

Response:

While we did not directly quantify the abundance of ATP synthase dimers (this is acknowledged in line 541), we quantified the abundance of ATP synthase monomers through clear-native PAGE (Fig. 4H) and the abundance of ATP5MK, a protein involved in promoting dimerisation of ATP synthase. The increased abundance of ATP synthase monomers, along with the increased abundance of ATP5MK (Fig. 4G) and increased cristae curvature (Fig. 2J-L), collectively suggest an increase in ATP synthase dimers in the cytotrophoblast.

We have now clarified this by amending the language in the discussion from referring to “ATP synthase dimers”, to now “the potential for ATP synthase dimerisation”.

Reviewer #1 (Remarks to the Author):

The authors have responded to many of the concerns raised in the review predominantly through textual clarification rather than through new experiments or analyses. While I appreciate the clarification and attempts to address concerns, such as the issues in delineating dimers versus monomers through subtomogram averaging efforts, without new information added to the manuscript, it's difficult to discern exactly what we have learned beyond what has been described in previous publications. The authors present differences in mitochondria from CTB and STB of the placenta. However, such differences have been noted previously using 2D imaging methods, as mentioned by the authors themselves. The additional in silico analysis of previously published proteomics data (from the same group) reveals differences in mitochondrial complex proteins between the two mitochondrial subtypes. However, similar observations have also already been presented in the previous publication. Without new information, either from mapping changes in macromolecular complexes or from differences in mitochondrial localization across these subtypes, it is hard to see how this paper advances our understanding of placental development beyond what has been shown previously.

Response:

We thank the reviewer for the additional opportunity to address their concerns.

We wish to acknowledge that further analysis had been performed in order to address previous concerns regarding the presentation of data points (Fig. 2 adjusted) and graphs separated by placenta (Supplementary Fig. 10). In addition to this, all array tomography data was reanalysed through a linear mixed effects model to account for cell and placental factors, which did not alter any significance between groups and only strengthened our confidence in the findings (Supplementary Fig. 4-9).

The application of subtomogram averaging to reveal the localisation and structure of protein complexes within cristae membranes, would be a great advancement. As previously stated, we believe this would be a manuscript in itself given the tremendous monetary and time investment required. We also wish to acknowledge that such an extensive methodology demands fluorescence-guided FIB-SEM lamellae milling, a method that has not yet been performed in the placental field, and has not been used to delineate mitochondrial subpopulations in neighbouring cells. Additionally, the collaborating regional institutes where we performed cryo-ET do not possess the equipment necessary for this methodology. This challenge is confounded by the major logistical difficulties of transporting samples in vapour nitrogen across large distances within Australia, to institutes with this capacity, which is not feasible to us at this time.

We continue to believe that our manuscript does present novel insights for both the placental, and the broader mitochondrial fields. With specific reference to the placental field, the traditional 2D analyses referred to by the reviewer fails to provide understanding of mitochondrial branching, showcased by our 3D reconstructions and quantitative analysis of mitochondrial complexity index (Fig. 1L). Further, 2D methods do not quantify any physiologically pertinent metrics such as mitochondrial surface area or volume, a finding that has only been suggested or speculative until this manuscript examined complex 3D topology and provided statistically proven differences between the CTB and STB mitochondria (Fig. 1G-L). We disagree that this study does not advance the field's understanding of placental

development, specifically since our study presents the first application of array tomography in the placental field and provides insights into the nature of villous structure and that CTBs are not purely "cuboidal underlying cells overlayed by the STB" as the 2D dogma has implied. When examined in the Z-dimension, we have shown that, infact, the STB and basement membrane is intricately interwoven around long cellular projections and processes of the CTB. This data is also now available for viewing as the array tomography data has been placed within the EBI BioImage Archive (Accession ID: S-BIAD3408) for full transparency.

Additionally, this study also represents the first application of cryo-ET in the placental field, leading to the interrogation of cristae structure with a resolution that is not currently possible with volume electron microscopy. Our proteomic data, while acquired in a previous study, has not been published, and like the field at large our discussion of these findings and differences was associative not quantitative. This proteomic data supports the changes in structure that we have observed and quantified in this study, suggesting mechanistic pathways that underpin mitochondrial remodelling for supporting cellular differentiation. Moreover, these findings were robustly assessed in additional and independent samples (n=7). More broadly, this study is the first to provide a subunit-specific assessment of the ETC complexes in Cyto- and Syncytio-Mitochondria, and associate this with higher supercomplex formation in the Cyto-Mitochondria through CN-PAGE (Fig 3, 4).

With specific reference to the broader mitochondrial field, we have applied the curvedness metric, and while this metric has been used to compare local cristae curvature within a single mitochondrion in other studies, to our knowledge it has not yet been used to compare cristae curvature between mitochondrial subpopulations. Our observations of curvedness demonstrate the capacity for mitochondrial remodelling and cristae differentiation to adapt their very architecture alongside protein-level changes, in order to support cellular differentiation.

Overall, we believe that even in the absence of subtomogram averaging, this study provides numerous novel advances to our understanding of mitochondrial dynamics, cristae architecture, the electron transport chain, and critical proteins involved in these processes. Indeed, our manuscript preprint has already been cited by a published review (https://doi.org/10.1016/j.jbc.2026.113174, Journal of Biological Chemistry), which discusses our paper's contribution of "biochemical analyses in accelerating cryo-ET studies of the MIB complex", and revealing "evolutionary adaptations linked to energetic demands". We believe that the dissemination of this work is crucial for filling a gap in our understanding of mitochondrial remodelling during cellular differentiation.

Reviewer #2 (Remarks to the Author):

I co-reviewed this manuscript with one of the reviewers who provided the listed reports. This is part of the Nature Communications initiative to facilitate training in peer review and to provide appropriate recognition for Early Career Researchers who co-review manuscripts.

Response:

We thank the reviewer for providing feedback on this latest revision of the manuscript.

Reviewer #3 (Remarks to the Author):

The manuscript has been revised in line with reviewers suggestions and is an impressive piece of work. I only have a few additional suggestions.

Response:

We thank the reviewer for acknowledging the revisions, the significance of this work, and for the additional suggestions.

Public accessibility of image data is important for future validation and reuse. In addition to inclusion within the source data file the raw image data figures should also be uploaded to a publicly available online repository such as bioimage archive (https://www.ebi.ac.uk/bioimage-archive/). These repositories represent a more searchable and accessible than accompanying data to a paper facilitating access and reuse. It is not clear why the image reconstructions could not also be uploaded here.

Response:

We have now deposited array tomography image series and model files on EBI BioImage Archive (Accession ID: S-BIAD3408). We have also deposited cryo-ET raw tilt series and tomograms on EBI BioImage Archive (Accession ID: S-BIAD3409). The accession codes have been added to the manuscript data availability statement, and to the Nature Reporting Summary checklist.

Line 150: “ideal model” is a bold claim and could be rephrased.

Response:

We have now adjusted this line to “The human placenta is a valuable model for studying mitochondrial diversity within a single tissue”.

In figure 1 I and L there are a couple of points with near zero values, out of keeping with the distribution of the rest of the data. Are the author’s certain these represent true mitochondria?

Response:

We thank the reviewer for raising this point, and have reviewed our data carefully. Upon inspection, the near-zero values belonged to two mitochondria that were excluded as outliers in the data, but these changes had not carried across to the graphs. We have now ensured that these values have been excluded from the graphs in Figure 1 and all analyses.

The authors could also consider referring to “mitos” as “mitochondria”

Response:

We have revised the manuscript and figures to refer to the populations as Cyto-Mitochondria and Syncytio-Mitochondria.

Reviewer #4 (Remarks to the Author):

The authors have addressed the majority of comments appropriately and the manuscript is now more easily readable. This reviewer however is still concerned by use of the term cyto-mito to describe the preparation used. Despite arguments that cytotrophoblast may comprise more than 10% of placental tissue at term the authors do not acknowledge that their cyto-mito preparation in all probability does contain mitochondria from other placental cell types including, endothelial cells, stroma, smooth muscle and macrophages, as they homogenize whole villous tissue. They present a robust defense of their preparation and cite other manuscripts in support. In examining these however the Kolahi et al 2017 paper does not use mitochondrial preparations, and the Martinez et al 1997 paper uses the technique only to prepare highly enriched syncytiotrophoblast mitochondria for study and does not study the remaining (cytotrophoblast + other) mitochondria from the preparation. The authors used the Martinez technique and alone invented the terminology cyto-mito but have never acknowledged the preparation probably contains more than cyto-mito. Statements about the number of citations do not negate this argument. Further arguments related to purity of preparations are an obfuscation here as purity of a mitochondrial preparation does not relate to derivation from different cell types. Can the authors present data showing the cellular origin of their preparation?

Response:

We thank the reviewer for acknowledging that appropriate revisions have been made to the manuscript, and for the opportunity to address these concerns.

To address the cellular origin of Syncytio-Mitochondria, the original protocol developed by *Martinez et al.*, showed that the STB-mitochondrial fraction displayed similar levels of $P450_{SCC}$-containing mitochondria as observed in situ within placental tissue, indicating the cellular origin of Syncytio-Mitochondria. However, P450scc is still present, albeit lower, in Cyto-Mitochondria, and therefore may not be a truly accurate marker of their cellular origin.

Given that an exclusive marker for either Cyto- or Syncytio-Mitochondria does not yet exist across imaging modalities, we have completed additional analysis comparing the transverse diameter of our Cyto- and Syncytio-Mitochondria observed via cryo-ET (Fig. 2), to their in situ counterparts quantified by array tomography (Fig. 1) to confirm their cellular origin.

Our additional analysis (shown below and as Supplementary Fig. 14) highlights that the isolated Cyto- and Syncytio-Mitochondria retain the expected transverse diameter distribution and capture the extent of heterogeneity observed within tissue, and are therefore structurally representative of their in situ counterparts observed via array tomography. The in situ CTB mitochondria (n=35) had a mean diameter of 510±88 nm, comparable to the isolated Cyto-Mitochondria observed by cryo-ET (n=8), which had a mean diameter of 539±54 nm. In situ STB mitochondria (n=35) had an expectedly lower mean diameter of 244±41 nm, which was also comparable with the isolated Syncytio-Mitochondria (n=8) diameter of 189±72 nm. We also performed this analysis on villous endothelial mitochondria and Hofbauer mitochondria (n=20 each), revealing a distinct structural divergence from both Cyto- and Syncytio-Mitochondrial populations. Endothelial mitochondria had a mean diameter of 288±54 nm, while Hofbauer mitochondria had a mean diameter of 428±71 nm. With reference to the reviewer's specific comments on the potential for contamination of Cyto-Mitochondrial isolation by other villous mitochondria, we observed 0% overlap of interquartile ranges between Cyto-Mitochondria and endothelial mitochondria, and only 2.2% overlap between

Cyto-Mitochondria and Hofbauer mitochondria. Although the diameter distributions between Syncytio-Mitochondria, Hofbauer, and endothelial mitochondria exhibited a small overlap, like CTB mitochondria in situ these mitochondria display distinct lamellar cristae morphology and thus can clearly be distinguished from the globular cristae of Syncytio-Mitochondria in Fig. 2 (cryo-ET). Collectively, this analysis enables us to ensure the cellular origin of mitochondria assessed as CTB and STB by cryo-ET, and are indeed representative of their in situ counterparts as indicated by the micrographs below and in Supplementary Fig. 14.

This additional analysis unifies our imaging modalities (array tomography and cryo-ET), and demonstrates that our isolated Cyto- and Syncytio-Mitochondria retain the expected structural hallmarks of their in situ counterparts, and do not exhibit the characteristics of villous endothelial or Hofbauer mitochondria. Therefore, this gives us great confidence that we have quantified exclusively CTB and STB mitochondria. In lieu of this analysis, we have also added the following to our limitations and methods sections "While we acknowledge that a minor contribution from other villous cell types cannot be completely excluded without an exclusive marker for the cellular origin of isolated mitochondrial populations, further analysis of structural data demonstrates that the isolated Cyto- and Syncytio-Mitochondria retain the expected transverse diameter distributions of their in situ counterparts observed via array tomography, and are structurally divergent from endothelial and Hofbauer mitochondria (Supplementary Fig. 14)." now line 603 and line 708.

We believe the capacity to perform this analysis further strengthens the manuscript's multimodal imaging approach and to the best of our current abilities accounts for cellular origin. We anticipate that new approaches for tissue-level cryo-ET will emerge in the near future, allowing for targeted and in situ high resolution structure interrogation, and are ourselves in the process of developing such methodologies.

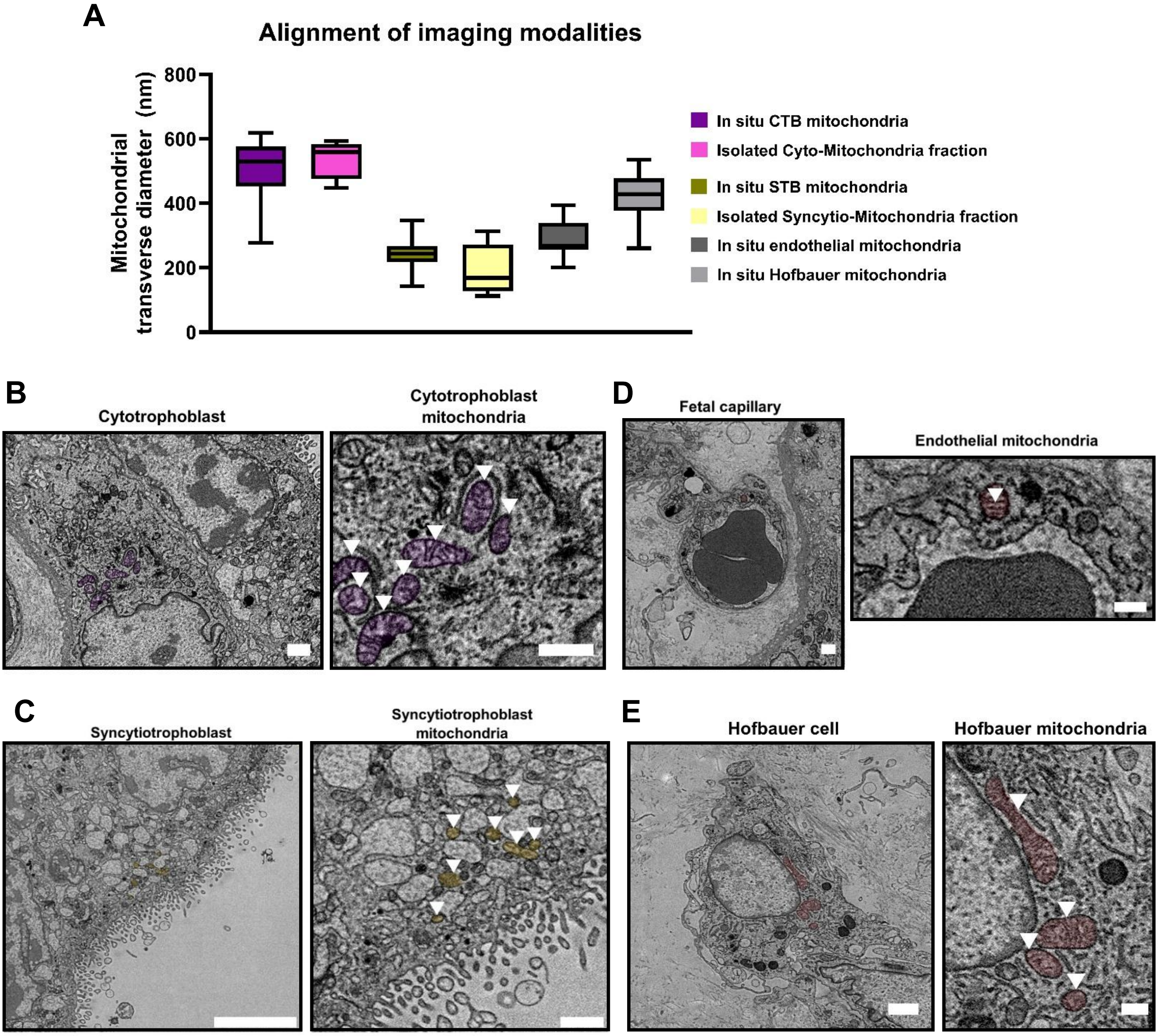

Supplementary Figure 14: Persistence of distinct mitochondrial phenotype following isolation. A) The graph demonstrates that isolated Cyto- and Syncytio-Mitochondria (cryo-ET) retain the expected diameter distributions of their in situ counterparts (array tomography). Interquartile ranges do not overlap across groups, and clear separation of central distributions between endothelial, Hofbauer, CTB, and STB mitochondria indicates the absence of structurally divergent and contaminating mitochondria within the mitochondrial isolations. In situ CTB mitochondria (n=35; purple), Isolated Cyto-Mitochondria fraction (n=8; pink), In situ STB mitochondria (n=35; dark yellow), Isolated Syncytio-Mitochondria fraction (n=8; light yellow), In situ endothelial mitochondria (n=20; dark grey), In situ Hofbauer mitochondria (n=20; light grey). Micrographs below the graph showcase a B) CTB cell with a magnified region of CTB mitochondria with variably orientated lamellar and tubulo-vesicular cristae (scale bars represent 1 μm); C) STB (scale bar represents 500 nm), with a magnified region of STB mitochondria (scale bar represents 100 nm); D) fetal capillary (scale bar represents 1 μm), with a magnified region of endothelial mitochondria with packed, aligned lamellar cristae (scale bar represents 500 nm); and E) Hofbauer cell (scale bar

represents 2 μm), with a magnified region of Hofbauer mitochondria with packed, aligned lamellar cristae (scale bar represents 500 nm).

Reviewer #4 (Remarks to the Author):

The imaging data supplied (array tomography and cryo-ET) clearly shows the distinction between cytotrophoblast and syncytiotrophoblast mitochondria and the supplemental data in Fig 14 shows distinction in size of mitochondria from hofbauer and endothelial cells in the preparation. The preparations used for the proteomic analysis and the immunoblot and western blot analyses likely however contain contributions from other villous tissue cell types. While the authors now acknowledge this limitation this may be more than "minor" as 60% of villous tissue volume is not trophoblast.

Response:

We thank the reviewer for acknowledging the supplemental analysis. We have now removed the word ‘minor’ from our limitations statement. In line with Editor’s recommendations, we have now amended all areas of text that refer to Cyto- or Syncytio-Mitochondria and have used isolations, to specify “preparations enriched for Cyto- or Syncytio-Mitochondria”, or “Cyto- or Syncytio-Mitochondria enrichments”.
